# Blood hsa-miR-122-5p and hsa-miR-885-5p levels associate with fatty liver and related lipoprotein metabolism—The Young Finns Study

**DOI:** 10.1038/srep38262

**Published:** 2016-12-05

**Authors:** Emma Raitoharju, Ilkka Seppälä, Leo-Pekka Lyytikäinen, Jorma Viikari, Mika Ala-Korpela, Pasi Soininen, Antti J. Kangas, Melanie Waldenberger, Norman Klopp, Thomas Illig, Jaana Leiviskä, Britt-Marie Loo, Niku Oksala, Mika Kähönen, Nina Hutri-Kähönen, Reijo Laaksonen, Olli Raitakari, Terho Lehtimäki

**Affiliations:** 1 Department of Clinical Chemistry, Pirkanmaa Hospital District, Fimlab Laboratories, and University of Tampere, School of Medicine, Tampere, Finland; 2 Division of Medicine Turku University Hospital and Department of Medicine, University of Turku, Turku, Finland; 3 Computational Medicine, Faculty of Medicine, University of Oulu and Biocenter Oulu, Oulu, Finland; 4 NMR Metabolomics Laboratory, School of Pharmacy, University of Eastern Finland, Kuopio, Finland; 5 Computational Medicine, School of Social and Community Medicine and the Medical Research Council Integrative Epidemiology Unit, University of Bristol, Bristol, UK; 6 Research Unit of Molecular Epidemiology, Helmholtz Zentrum, German Research Center for Environmental Health, Munich, Germany; 7 Hannover Unified Biobank, Hannover Medical School, Hannover, Germany; 8 Institute for Human Genetics, Hannover Medical School, Hanover, Germany; 9 Department of Health, National Institute for Health and Welfare, Helsinki and Turku, Finland; 10 Division of Vascular Surgery, Department of Surgery, Tampere University Hospital, Tampere, Finland; 11 Department of Clinical Physiology, Tampere University Hospital, and School of Medicine, University of Tampere, Tampere, Finland; 12 Department of Pediatrics, University of Tampere and Tampere University Hospital, Tampere, Finland; 13 Research Centre for Applied and Preventive Cardiovascular Medicine, University of Turku, Turku, Finland; 14 Department of Clinical Physiology and Nuclear Medicine, University of Turku and Turku University Hospital, Turku, Finland

## Abstract

MicroRNAs are involved in disease development and may be utilized as biomarkers. We investigated the association of blood miRNA levels and **a**) fatty liver (FL), **b**) lipoprotein and lipid pathways involved in liver lipid accumulation and **c**) levels of predicted mRNA targets in general population based cohort. Blood microRNA profiling (TaqMan OpenArray), genome-wide gene expression arrays and nuclear magnetic resonance metabolomics were performed for Young Finns Study participants aged 34–49 years (n = 871). Liver fat status was assessed ultrasonographically. Levels of hsa-miR-122-5p and -885-5p were up-regulated in individuals with FL (fold change (FC) = 1.55, p = 1.36 * 10^−14^ and FC = 1.25, p = 4.86 * 10^−4^, respectively). In regression model adjusted with age, sex and BMI, hsa-miR-122-5p and -885-5p predicted FL (OR = 2.07, p = 1.29 * 10^−8^ and OR = 1.41, p = 0.002, respectively). Together hsa-miR-122-5p and -885-5p slightly improved the detection of FL beyond established risk factors. These miRNAs may be associated with FL formation through the regulation of lipoprotein metabolism as hsa-miR-122-5p levels associated with small VLDL, IDL, and large LDL lipoprotein subclass components, while hsa-miR-885-5p levels associated inversely with XL HDL cholesterol levels. Hsa-miR-885-5p levels correlated inversely with oxysterol-binding protein 2 (OSBPL2) expression (r = −0.143, p = 1.00 * 10^−4^) and suppressing the expression of this lipid receptor and sterol transporter could link hsa-miR-885-5p with HDL cholesterol levels.

Fatty liver (FL) is characterized by the accumulation of triacylglycerol-rich lipid droplets in hepatocytes (liver fat >5–10% of liver weight). The primary causes leading to hepatocellular lipid accumulation are not yet well understood, but they are thought to include alterations in the hepatic lipid uptake, synthesis, degradation, and secretion^1^. FL is the first stage of a continuum from benign, simple steatosis to steatohepatitis, fibrosis, cirrhosis, and even hepatocellular carcinoma^2^. Non-alcoholic FL disease (NAFLD) is defined as FL without evidence of excessive alcohol intake or other causes of steatosis^2^ and it has histological and pathological similarities with alcoholic liver disease (ALD)^3^. NAFLD is common in Western and Asian populations with a prevalence of 20–30% in the general population and 70–90% among the obese and diabetics^4^. It is also frequently associated with obesity, type 2 diabetes, and hyperlipidemia^4^. Individuals with ALD have been shown to be as metabolically unhealthy as subjects with NAFLD, and it is highly possible that ALD and NAFLD coexist^5^. In our population based Young Finns Study (YFS) sample, we have recently observed that alcohol consumption was significantly associated with the prevalence of FL only in normal-weight individuals, whereas in overweight or obese subjects the most significant correlates of FL were body mass index (BMI) and triglycerides^6^. Majority of the individuals with FL in YFS (ages 34–49 years) are men (74.1%). This is to be expected, as even though overall prevalence of NAFLD has been shown to be similar between the sexes, men reach their peak prevalence of NAFLD in their forties, whilst in women the prevalence of NAFLD is still increasing and reaches its peak in their sixth decade^7^. Although women are known to develop ALD with lesser alcohol consumption, men in YFS consume considerable more alcohol than the women^8^.

MicroRNAs (miRNAs, miRs) are small non-coding RNAs that primarily regulate gene expression by binding to target mRNAs and interfering with their translation^9^. MicroRNAs are transcribed from DNA to primary transcripts, which are then cleaved to shorter hairpin structures, exported to cytoplasm, and further processed to form mature one-stranded miRNAs. In most cases, miRNAs repress their targets by interaction with the 3′UTR of the target mRNA, inducing a detectable change in the mRNA levels^10^. MicroRNAs can also be transported between cells and tissues via circulation. Membrane-free miRNAs stabilized by proteins^11^,^12^ and miRNAs associated with lipid vesicles can be found in blood. Circulating miRNAs have also been found in high-density lipoproteins (HDL)^13^,^14^. Circulating miRNAs have been shown to participate in cell-to-cell communication^15^, potentially contributing to disease progression. Humans are predicted to have 2,588 mature microRNAs (mirBase, release 21) that can control several genes, and individual mRNAs can be bound by several miRNAs—miRNAs can thus establish wide regulatory networks affecting several metabolic processes.

The development of FL has been shown to alter the miRNA profiles in the liver^16^,^17^, serum^18^,^19^,^20^, and exosomes^21^. MicroRNAs have also been shown to affect the regulation of cholesterol metabolism^22^, liver inflammation^23^, hepatic fibrosis^24^, and the development of hepatocellular carcinoma^25^, all associated with NAFLD and ALD^26^,^27^. Even a serum miRNA expression panel consisting of hsa-miR-122-5p, -1290, -27b-3p, and -192-5p has been suggested for clinical NAFLD diagnostics^18^. Studies have shown increased levels of circulatory miR-122 in liver diseases with different etiologies^18^,^20^,^23^,^28^,^29^,^30^,^31^, indicating that it may be a potential marker for liver injury. In addition, for example the circulatory miRNAs related to inflammation, such as miR-21, -34a, -451^28^ and -155 have been associated to NAFLD (reviewed in refs 32 and 33). Still, many of the studies of miRNAs in FL disease have been performed with animal models. So far, miRNA profiling in humans has been done in small selected groups, and the larger studies have focused only on few specific preselected miRNAs^18^,^19^,^20^,^21^,^23^,^28^,^30^,^34^. These studies have also been performed in non-population based cohorts, and thus cannot be directly applied to general population. Therefore, wider miRNA profiling analyses with an adequate sample size and population based sampling are needed. The YFS is well-suited for this profiling, as the study participants have been extensively characterized and the population has a very low prevalence of viral hepatitis: according to the Finnish Red Cross, the prevalence of hepatitis B and C was 0.03% and 0.05%, respectively, among Finnish blood donors^5^.

The aims of the current study were to (***i***) compare blood miRNA levels between individuals with or without FL in a large population-based study cohort, (***ii***) investigate the association of discovered miRNAs and liver enzyme levels and explore the predictive ability of these miRNAs to detect FL over established risk factors, (***iii***) test the hypothesis that these miRNAs might be involved in the regulation of lipoprotein and lipid pathways connected to liver lipid accumulation by studying the association between dysregulated miRNAs and nuclear magnetic resonance (NMR) metabolomics and other characteristic previously associated with a metabolic dysfunction, (***iv***) further explore the mode of function of the miRNAs by analyzing the connections of these FL associated miRNAs with their predicted mRNA targets using transcriptomics analyses from the same samples. The flow and summary of the study is described in Supplementary Figure 1.

## Materials and Methods

### The Young Finns Study

YFS is a multicenter follow-up study on cardiovascular risk from childhood to adulthood in Finland. The YFS was launched in 1980, when 3,596 children and adolescents (3–18 years old) participated in the baseline study^35^. The subjects were randomly selected from Finnish national registry among the chosen age groups and the five study districts. Thereafter, the subjects have been followed up (in 1986, 2001, 2007 and 2011) with several examinations including comprehensive risk factor assessments. The 30-year follow-up was performed in 2011, with 2,063 adults, aged 34–49 years, participating in the study. The examinations included physical measurements, blood tests, and questionnaires. Participants in the follow-up studies have been found to be more often women and older than those who dropped out, but no significant differences in risk factors have been found^35^. The present study has been approved by the 1st ethical committee of the Hospital District of Southwest Finland on September 21st, 2010 and by local ethical committees. All study subjects gave an informed consent and the study was conducted according to the principles of the Declaration of Helsinki. YFS samples for miRNA analysis (n = 992) were selected independently form liver status from individuals having the most comprehensive data on risk factor, metabonomic, transcriptomics, genome wide genotyping and other phenotyping in the follow-up studies performed in 1986, 2001, 2007 and 2011. After quality control, study population comprises of 871 individuals with successful miRNA profiling (demographics in Table 1).

### Clinical and biochemical measurements

Weight and height were measured and BMI calculated as weight(kg)/(height(m))^2^. Waist circumference was measured to the nearest 0.1 cm. Blood pressure was measured with a random zero sphygmomanometer.

Venous blood samples were drawn from the right antecubital vein after an overnight fast. For blood count analysis, whole blood was anticoagulated with EDTA. Blood cell parameters were measured by flow cytometric particle counting (cells) and photometry (Hb) using Sysmex XE- 5000 and XT-2000i analyzers (Sysmex Corporation) with reagents provided by the manufacturer (Cellpack and Sulfolyser).

For the biochemical measurements, serum was separated, aliquoted and stored at −70 °C until analysis. Serum alanine aminotransferase (ALT), aspartate aminotransferase (AST), gamma-glutamyl transferase (GT), glucose, cholesterol, and triglyceride concentrations were measured with ALT, AST, GT, Glucose, Cholesterol, and Triglycerides System Reagent, (Beckman Coulter Biomedical). Apolipoprotein A1 (ApoA1), apolipoprotein B (ApoB), and C-reactive protein (CRP) were determined immunoturbidimetrically (ApoA1 and B assay reagent, Orion Diagnostica and CRP Latex reagent, Beckman Coulter Biomedical). The serum triglyceride concentration was assayed using the enzymatic glycerol kinase–glycerol phosphate oxidase method (Beckman Coulter Biomedical). Serum total cholesterol levels were measured by the enzymatic cholesterol esterase–cholesterol oxidase method (Beckman Coulter Biomedical). The same reagent was used for estimating HDL cholesterol levels after the precipitation of low-density lipoprotein (LDL) and very low-density lipoprotein (VLDL) with dextran sulfate- Mg^2+^. Serum glucose concentrations were determined by the enzymatic hexokinase method (Beckman Coulter Biomedical). All the above-mentioned assays were performed on an AU400 instrument (AU400, Olympus).

Glycated hemoglobin (HbA1c) fraction in whole blood was measured by an Abbott Architect ci8200 analyzer (Abbott Laboratories). The concentration of total hemoglobin was first determined colorimetrically, after which the concentration of HbA1c was measured immunoturbidimetrically using the microparticle agglutination inhibition method (Fisher Diagnostics). These two concentrations were used to calculate the HbA1c percentage. All the above-mentioned methods besides ALT, AST, and GT quantifications were accredited by the Finnish Accreditation Service (FINAS).

### NMR metabolomics

A high-throughput serum NMR metabolomics platform was used for absolute quantification of serum lipids and metabolites, including lipoprotein subclass distributions, fatty acids, and various small molecules such as amino acids and glycolysis precursors^36^,^37^ (Supplementary Table 1). The analyzed 14 lipoprotein subclasses were defined based on particle size. These detailed lipoprotein subclass measurements, together with standard total lipids and apolipoproteins, provide a good basis for studying the lipid- and lipoprotein-related metabolic pathways. The NMR-based metabolic profiling has previously been used in various epidemiological and genetic studies^37^, and details of the experimentation have been described^36^,^37^,^38^. Data was available from all the individuals with successful miRNA profiling.

### Liver ultrasonography

Ultrasound imaging and evaluation of hepatic fat in hepatic ultrasound scan images (taken in 2011) of the liver was performed using a validated protocol^39^ and Sequoia 512 ultrasound mainframes (Acuson) with 4.0 MHz adult abdominal transducers. The diagnostic evaluation of hepatic steatosis was performed visually using a standard system by a highly trained sonographer according to liver-to-kidney contrast, parenchymal brightness, deep beam attenuation, and bright vessel walls^40^. According to these criteria, the presence of hepatic steatosis (=fatty liver, FL_All_) was assessed and the participants were further classified into subjects with clearly identifiably fatty liver (FL_C_) or mild fatty liver (FL_M_), and normal liver (NL).

### RNA isolation and quality control

Whole blood (2.5 ml) was collected into PaXgene Blood RNA Tubes (PreAnalytix). The tubes were inverted 8–10 times then stored at room temperature for at least 2 hours. The tubes were frozen (−80 °C) and thawed overnight before RNA isolation (both miRNA and total RNA) with a PAXgene Blood microRNA Kit (Qiagen) including the DNase Set using the QiaCube. The concentrations and purity of the RNA samples were evaluated spectrophotometrically (BioPhotomer, Eppendorf). The RNA isolation process was validated by analyzing the integrity of several RNAs with the RNA 6000 Nano Chip Kit (Agilent). The presence of the small RNA fraction was confirmed by the Agilent Small RNA Kit (Agilent).

### MicroRNA expression profiling

MicroRNA expression profiling was performed with the TaqMan^®^ OpenArray^®^ MicroRNA Panel (Applied Biosystems) containing 758 microRNAs. Briefly, 100 ng of RNA was used to run both A and B pools of Megaplex (Applied biosystems) preamplification for cDNA synthesis. In the OpenArray Sample Loading Plate, 22.5 μl of each preamplified pool was mixed 1:1 with TaqMan OpenArray Real-Time PCR Master Mix. MicroRNA panels were loaded using the AccuFill System and run with the QuantStudio 12 K Flex (Applied Biosystems).

Primary data analysis was performed with Expression Suite Software version 1.0.1. As recommended by the manufactures of the miRNA panels, RNU6, RNU44, and RNU48 were used as housekeeping small RNAs. Assays with Amplification score >1 and Cq Confidence >0.7 were accepted. Ninety-five samples were excluded due to a low number of miRNAs expressed (≤200 miRNAs per sample), and in further analysis, 243 miRNAs that were expressed in at least 2/3 of the samples were included (number of miRNAs present in detectable levels in majority of the blood samples is well in line with previous similar analysis of blood tissue^41^). The RNA quality and functionality of the TaqMan OpenArray microRNA expression panels have been validated previously^42^. After quality control and removal of outlier miRNAs, profiling was successful on 871 samples. To correct for batch effects, the principal component analysis was performed for the miRNA expression data. The data was adjusted for 10 of the first 20 principal components from the principal component analysis.

### Genome-wide expression analysis (transcriptomics)

The expression levels were analyzed with an Illumina HumanHT-12 version 4 Expression BeadChip (Illumina Inc.). Utilizing the same RNA sample for both mRNA and miRNA expression profiling, 200 ng of RNA was reverse-transcribed into cDNA and biotin-UTP-labeled using the Illumina TotalPrep RNA Amplification Kit (Ambion); 1,500 ng of cDNA was then hybridized to the Illumina HumanHT-12 v4 Expression BeadChip. The BeadChips were scanned with the Illumina iScan system. Raw illumina probe data was exported from Genomestudio and analyzed in R (http://www.r-project.org/) using the Bioconductor (http://www.bioconductor.org/) packages. The expression data was processed using nonparametric background correction, followed by quantile normalization with control and expression probes, using the neqc function in the limma package and log2 transformation. Data processing described in more detailed in ref. 43. The expression analysis was successful in 743 of the 871 samples with a miRNA expression profile.

### Statistical analysis

MicroRNA expressions were compared over individuals with NL, FL_M_ and FL_C_ (using one-way ANOVA for normally distributed miRNAs). In order to take account of the multiple testing Bonferroni corrected p-values (p_c_-value) were calculated and p_c_-value < 0.05 (=p < 0.00021) was considered significant. For dysregulated miRNAs fold changes (FCs) were calculated for each individual sample in comparison to the average of all individuals with NL. FL index was calculated as previously described^44^ and the levels of discovered miRNAs were correlated with this index using Spearman’s rank-order correlation and independent association was evaluated with linear regression model adjusted with age and sex.

The independent predictors of fatty liver status (FL_All_ vs. NL and FL_C_ vs. NL) were researched using stepwise Akaike information criterion (AIC) logistic regression. Three different models were used as follows: Model 1: FL status (FL_All_/FL_C_ vs. NL) predicted with discovered miRNAs only, (one by one forced in to the model); Model 2: age, sex and BMI added among explanatory variables of model 1; and fully adjusted Model 3 including all explanatory variables known to be associated with FL in YFS study^6^ i.e., age, sex, BMI, alcohol consumption, waist circumference, age, apoB levels, triglycerides, insulin levels, systolic blood pressure, smoking, and physical activity index, (excluding liver enzymes due to high correlation with studied miRNAs). Only independent predictors of liver status remained in the final model. Similar analysis were done separately for normal weight (BMI < 25) and overweight or obese individuals (BMI > 25) as the explanatory parameters associated to FL have been shown to differ in these subpopulations^6^ and also separately for men and women and individuals with or without excess of alcohol consumption (>1.67 standard drinks per day; corresponding to 20 g of pure ethanol). In stratified analysis, only models predicting FL_All_ vs NL were possible, due to low number of individuals with FL_C_.

The sensitivity and specificity of the discovered miRNAs to detect individuals with FL_All_ or FL_C_ was analyzed by the receiver operator curve (ROC) analysis and areas under curve (AUC) were compared between miRNAs and the liver enzymes ALT, AST and GT. To evaluate the incremental predictive value of miRNA levels in comparison to parameters known to be associated with FL in YFS participants^6^ (liver enzymes, sex, BMI, waist circumference, age, apoB levels, triglycerides, insulin levels, systolic blood pressure, smoking, alcohol consumption, and physical activity index), a continuous net classification improvement (NRI) was calculated using reclassification function in the PredictABEL R package. Only parameters with independent association to liver status were included in the model (ALT, GT, waist circumference, insulin levels and systolic blood pressure). All continuous variables were inverse-normalized. A p-value < 0.05 for continuous NRI was considered significant.

The correlation between miRNAs and metabolite levels as well as physiological features previously associated with metabolic dysfunction (list of metabolites and other characteristics shown in Supplementary Table 1) was analyzed using Spearman rank-order correlation. The independent predictors value of the FL associated miRNAs was analyzed by applying the stepwise AIC regression model including FL associated miRNAs (one by one forced into model) and sex, age, BMI, and liver status as explanatory variables when analyzing liver enzymes, and also with ALT, AST and GT when analyzing other phenotypes. All continuous variables were inverse-normalized, and individuals with ALT, AST or GT levels over the Finnish reference ranges were discarded from the analysis when other phenotypes were used as an dependent variable in the model.

The predicted mRNA target expressions were included in the analysis if they were recognized as a target of the miRNA of interest by at least two target prediction programs in miRGator v.3.0^45^. Spearman’s rank-order correlations between the FCs of the miRNAs of interest and the expression their targets mRNA in transcriptomics analysis with a p < 0.05 are reported. An independent association was assessed with a same model as with metabolites. The pathways enriched by the down-regulated (p < 0.05) predicted target of these microRNAs were discovered by computing overlaps in a molecular signature database (http://www.broadinstitute.org/gsea/msigdb/annotate.jsp).

## Results

### Differences in blood levels of hsa-miR-122-5p and hsa-miR-885-5p between individuals with and without FL

**Figure 1:**
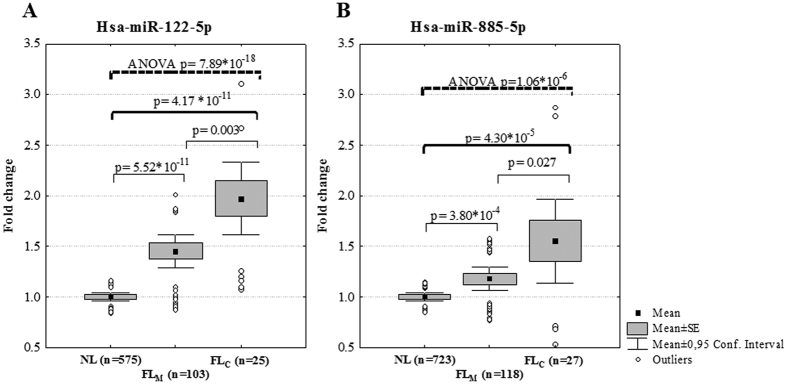
Blood levels of hsa-miR-122-5p (**A**) and hsa-miR-885-5p (**B**) in individuals without fatty liver (NL), or with mild (FL_M_) or clearly indentified fatty liver (FL_C_). Abbreviations: ANOVA = Analysis of variance, SE = standard error.

One-way ANOVA over liver fat status groups (NL, FL_M_ and FL_C_) showed a significant increasing trend for the levels of hsa-miR-122-5p (assay number 002245, successfully profiled form 703 individuals, p = 7.89 * 10^−18^, Bonferroni corrected p_c_ = 1.92 * 10^−15^) and hsa-miR-885-5p (assay number 002296, successfully profiled form 868 individuals, p = 1.06 * 10^−06^, p_c_ = 2.58 * 10^−04^). In comparison to individuals with NL, the expression of hsa-miR-122-5p was up-regulated in individuals with either FL_M_ (FC = 1.45, p = 5.52 * 10^−11^) or FL_C_ (FC = 1.97, p = 4.17 * 10^−11^) (Fig. 1A). Similarly, the expression of hsa-miR-885-5p was up-regulated in individuals with FL_M_ (FC = 1.18, p = 3.80 * 10^−4^) and in those with FL_C_ (FC = 1.55, p = 4.30 * 10^−5^) when compared to those with NL (Fig. 1B). Both, hsa-miR-122-5p and -885-5p were also up-regulated when comparing individuals with FL_All_ to individuals with NL (FC = 1.55, p = 1.36 * 10^−14^ and FC = 1.25, p = 4.86 * 10^−4^, respectively). These miRNAs were also associated with a FL, when analyzing separately in individuals with and without excess alcohol consumption (Supplementary Figure 2, One-way ANOVA over liver fat status groups; hsa-miR-122-5p p = 0.0001 and p = 3.10 * 10^−12^, respectively and hsa-miR-885-5p p = 0.005 and p = 0.007, respectively) to represent ALD and NAFLD.

There were sex*miRNA interaction with hsa-miR-122-5p and hsa-miR-885-5 in respect to presence of FL_All_ (p = 2.69 * 10^−4^ and 0.046, respectively). In men the association of hsa-miR-122-5p levels with FL status (NL vs. FL_M_ vs. FL_C_) was in parallel with women but statistically stronger (p = 1.72 * 10^−12^ for trend) than in women (p = 0.001). In similar analysis for hsa-miR-885-5p levels, the differences over liver status groups was seen only in men (p = 0.001 for men and p = 0.121 for women). Hsa-miR-122-5p and 885-5p correlated with FL index (r = 0.281, p = 3.54 * 10^−14^ and r = 0.105, p = 0.002, respectively). In linear regression model including age, sex and miRNA, hsa-miR-122-5p remained a significant independent predictor of FL index (p = 4.16 * 10^−11^, β = 0.227, 95% CI = 0.161–0.294 SD change in FL index per one SD increase of miRNA levels).

### Hsa-miR-122-5p and -885-5p as predictors of fatty liver

In stepwise logistic regression model adjusted with age, sex and BMI hsa-miR-122-5p significantly predicted the existence of FL_All_ (OR = 2.07, 95% CI = 1.62–2.68, p = 1.29 * 10^−8^) and the effect of hsa-miR-122-5p remained significant also in the fully adjusted model (OR = 1.78 95% CI = 1.35–2.38, p = 6.84 * 10^−5^) (Table 2). In stratified analysis, with normal weight individuals (BMI ≤ 25) or overweigh and obese individuals (BMI > 25) the corresponding ORs were 3.1 (95% CI = 1.54–7.0, p = 0.003) and 1.57 (95% CI = 1.17–2.13, p = 0.003), respectively (supplementary Table 2). Similar ORs could also be seen when analyzing women and men separately (OR = 2.02, 95% CI = 1.20–3.56, p = 0.010 and OR = 1.77, 95% CI = 1.26–2.51, p = 0.001, respectively) and in moderate (<20 g alcohol per day) and excess alcohol users (≥20 g alcohol per day) (OR = 1.75, 95% CI = 1.28–2.44, p = 0.001 and OR = 2.03, 95% CI = 1.02–4.59, p = 0.059, respectively) (Supplementary Table 2).

Also hsa-miR-885-5p significantly predicted the existence of FL_All_ in stepwise logistic regression model adjusted with age, sex and BMI (OR = 1.41 95%CI = 1.13–1.77, p = 0.002). In the fully adjusted model (Model 3) hsa-miR-885-5p predicted FL_All_ when all other risk factors were added to the model (including alcohol consumption), but the addition of insulin levels abolished the significance of the predictive value of this miRNA (OR = 1.23 95%CI = 0.94–1.60, p = 0.131) (Table 2). In the sex, weight and alcohol usage stratified analysis, hsa-miR-885-5p was an independent predictor of FL_All_ in the model including age, BMI and sex (no included in the sex stratified analysis) but not in the fully adjusted model, with the exception of the group of subjects with excess alcohol usage where the results remained significant (OR = 2.08, 95%CI = 1.18–3.94, p = 0.016) (Supplementary Table 2).

### Associations of hsa-miR-122-5p and -855-5p with serum liver enzymes

Hsa-miR-122-5p and 855-5p both correlated with the levels of ALT, AST and GT (p_c_ < 3.05 * 10^−6^ for all). Also in stepwise linear regression models including age, sex, BMI and liver status, the individual FL associated miRNAs (hsa-miR-122-5p and -885-5p one by one) predicted significantly all liver enzyme levels (Table 3).

### Improvement of fatty liver prediction by hsa-miR-122-5p and -855-5p over traditional fatty liver risk factors

**Figure 2:**
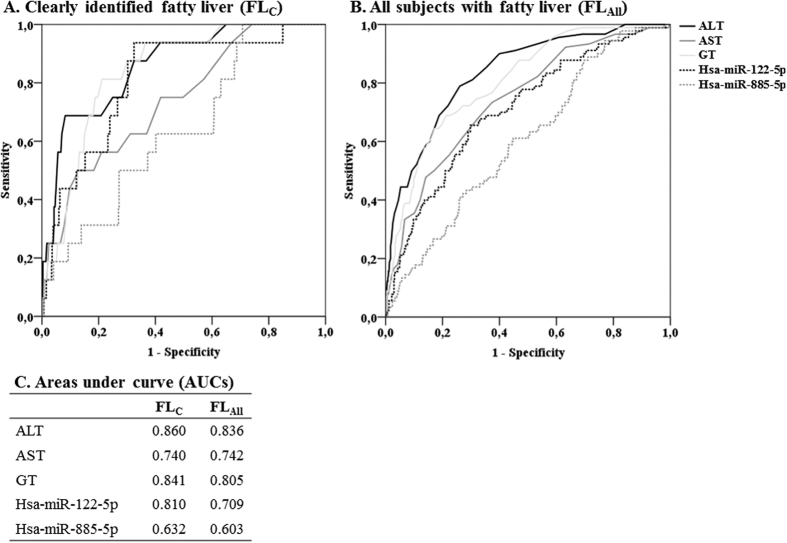
Receiver operating characteristi curve (ROC) analysis of the clinical utility (sepecificity and sensitivity) of blood levels of hsa-miR-122-5p and -885-5p in comparison to the serum levels of liver enzymes when predicting individuals with FL_C_ (**A** and **C**) or FL_All_ (**B** and **C**). Abbreviations: ALT = alanine aminotransferase, AST = aspartate aminotransferase, GT = gamma-glutamyltransferase, FL_All_ = all subjects with fatty liver, FL_C_ = clearly identified fatty liver.

When comparing the utility of FL associated miRNAs in identifying individuals FL_C_ or with FL_All_ to commonly utilized liver enzyme levels (ALT, AST and GT), the AUC of the hsa-miR-122-5p levels from the was comparable to ALT and GT (Fig. 2A–C). Moreover, hsa-miR-122-5p outperformed AST when detecting individuals with FL_C_ (Fig. 2A and C). In this ROC analysis when comparing AUCs of hsa-miR-885-5p levels to those of liver enzymes in detecting FL_All_ all liver enzymes performed better than hsa-miR-885-5p (Fig. 2B and C).

Together hsa-miR-122-5p and -885-5p slightly improved the detection of FL_C_ beyond established risk factors alone (continuous NRI = 1.36, 95% CI = 1.06–1.67, p = 2.8 * 10^−18^) (Table 4). Also including these miRNAs to the model predicting individuals with FL_All_ showed minor, but significant NRI (NRI = 0.278, 95%CI = 0.077–0.480, p = 0.0067) (Table 4). In sex stratified analysis, hsa-miR-122-5p and -885-5p slightly improved the detection of FL_All_ beyond established risk factors alone in both men and women separately (continuous NRI = 0.445, 95% CI = 0.068–0.821, p = 0.021 for women and continuous NRI = 0.383, 95% CI = 0.142–0.624, p = 0.0019, for men) but not in the weight stratified analysis (Supplementary Table 3).

### Hsa-miR-122-5p and -885-5p as predictors of serum lipoprotein subclass components involved in lipid and lipoprotein pathways

**Figure 3:**
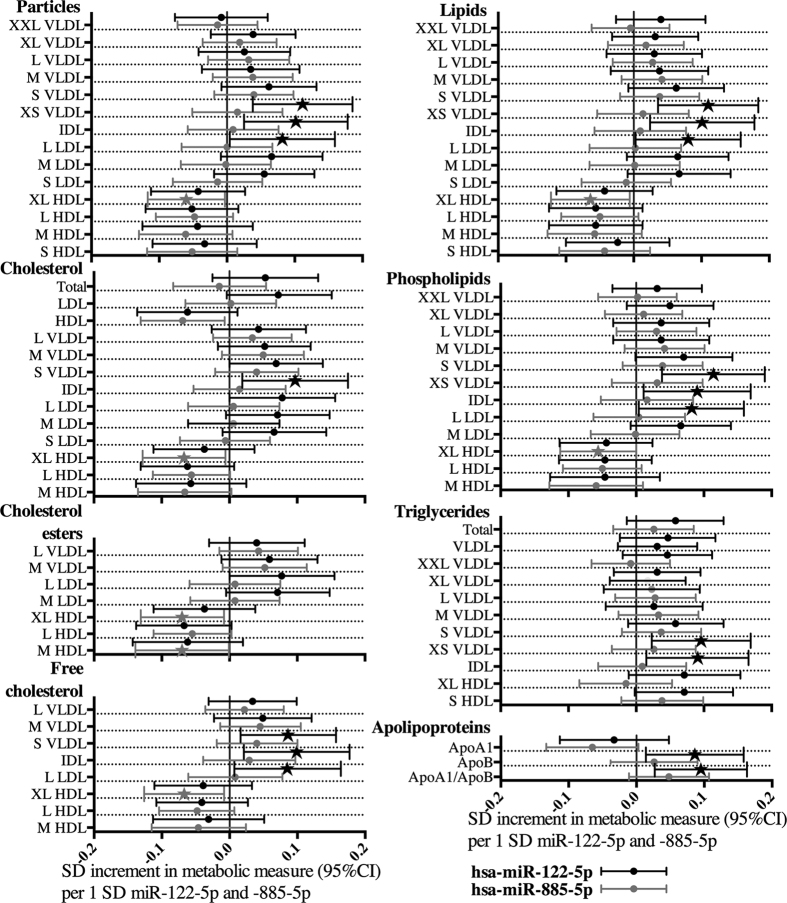
Associations of hsa-miR-122-5p and -885-5p with lipoprotein subclass particle size and lipid components as well apoliporoteins A1 and B. Standard deviation (SD) increment in metabolite measure and 95% confidence intervals (95% CI) per one SD change of miRNA are presented. Statistically significant results are highlighted with a star. Associations have been analyzed with a stepwise linear regression model adjusted with age, sex, BMI, liver status, alanine aminotransferase (ALT), aspartate aminotransferase (AST) and gamma-glutamyltransferase (GT) and individuals with ALT, AST or GT levels over the Finnish reference ranges have been removed from the analysis. Abbreviations: XL = extra large, L = large, M = medium, S = small, XS = extra small, HDL = high-density lipoprotein, LDL = low-density lipoprotein, IDL = intermediate-density lipoprotein, VLDL = very-low-density lipoprotein.

In metabonomics analysis, hsa-miR-122-5p and -885-5p levels correlated with several metabolites and physiological features associated with metabolic dysfunction (data not shown), but independent prediction value in linear regression model including sex, age, BMI, liver status, ALT, AST, GT and miRNA (miRNAs forced in the model one by one) existed only when predicting size and components of lipoprotein subclasses and apolipoprotein levels (Fig. 3, Supplementary Table 4). Hsa-miR-122-5p (n = 668) predicted small VLDL, IDL, and large LDL particle concentrations, their lipid component and cholesterol concentrations and apoB levels (Fig. 3). Hsa-miR-885-5p (n = 835) predicted significantly very large HDL subclass free cholesterol, cholesterol esters, total cholesterol, phospholipid and total lipid concentrations (Fig. 3. and Supplementary Table 4). The hsa-miR-122-5p also significantly predicted immunoturbidimetrically measured apoB levels (β = 0.092, 95%CI = 0.021–0.162, p = 0.011). Neither hsa-miR-122-5p nor -885-5p levels correlated significantly with leucocyte count (data not shown).

### Hsa-miR-122-5p and -855-5p as predictors of their in silico predicted target mRNA levels

Correlation and adjusted (age, sex, BMI, liver status and liver enzyme levels) linear regressions between hsa-miR-122-5p and -885-5p and *in silico* predicted mRNA target expression levels from transcriptomics analyses are shown in Table 5. Hsa-miR-885-5p levels correlated significantly with the levels of 48 predicted mRNA targets (Supplementary Table 5). The correlations with AHNAK nucleoprotein (AHNAK), trophinin (TRO) and oxysterol binding protein like 2 (OSBPL2) were significant after multiple testing correction (p_c_ < 0.05 i.e. p < 0.00021). In linear regression adjusted for age, sex, BMI, liver status, ALT, AST and GT, hsa-miR-885-5p predicted inversely (indicating a possible direct miRNA-induced target gene expression reduction) with GABA(A) receptor-associated protein (GABARAP), OSBPL2, arylsulfatase A (ARSA), and radial spoke 3 homolog (*Chlamydomonas*) (RSPH3) (Table 5), OSBPL2 being the only mRNA with statistically significant negative Spearman correlation with hsa-miR-885-5p levels (after multiple testing correction), of which levels were independently predicted by hsa-miR-885-5p levels in the regression model.

The majority of the 77 correlations (Spearman correlation p < 0.05) between hsa-miR-122-5p levels and its predicted target mRNA expressions levels were direct (Supplementary Table 6) and none were significant after multiple testing correction (p_c_ < 0.05 i.e. p < 0.00021). In linear regression model adjusted with age, sex, BMI, liver status, ALT, AST and GT, hsa-miR-122-5p inversely predicted eukaryotic translation initiation factor 1A, the X-linked (EIF1AX) and zinc finger CCCH-type containing 10 (ZC3H10) expressions, indicating their possible direct miRNA-induced target gene expression reduction (Table 5). In pathway analysis, miRNA targets with negative association to hsa-miR-122-5p and/or -855-5p were not enriched in any specific pathways.

## Discussion

We found that elevated blood levels of hsa-miR-122-5p and -885-5p were associated with ultrasonographically detected FL in individuals with and without excess alcohol consumption in a large, population-based cohort of adults. These miRNAs highly correlated with serum liver enzyme levels and FL index. In the prediction of FL_C_, the levels of hsa-miR-122-5p were comparable to most commonly used liver enzyme levels and both hsa-miR-122-5p and -885-5p together improved risk stratification beyond established risk factors. In the metabolomic analysis, hsa-miR-122-5p levels were found to independently predict apoB, small VLD, IDL, and large LDL component levels, while hsa-miR-885-5p levels were inversely associated with very large HDL total lipid, phospholipid and cholesterol content. Finally, the levels of hsa-miR-885-5p correlated inversely with its predicted target mRNA OSBPL2 expression, presenting a possible link between hsa-miR-885-5p and both HDL levels and it lipid content.

The association of hsa-miR-122-5p blood levels with FL, FL index and liver enzyme levels independently of age, sex, and BMI was shown here in a large clinically asymptomatic population. Levels of this miRNA remained as a significant predictor of FL when adjusting with known traditional risk factors of FL and the predictive value could be seen in normal weight and overweight individuals independently. This confirms the previous result where elevated miR-122 serum/plasma levels in individuals with NAFLD in comparison to those without has been detected small case-control profiling studies (n < 50)^18^,^20^,34 and two large expression analyses with selected miRNAs (n~400 each; 4–5 miRNAs quantified)^18^,^28^. Our study also extends the data applicability to general population. Hsa-miR-122-5p is almost solely expressed in the liver, and it is the most expressed miRNA in the liver, constituting approximately 70% of liver miRNA expression^46^. During the progression of NAFLD, liver miR-122 levels have been shown to be down-regulated in mice^47^,^48^ and humans^16^. There have also been conflicting results on the effects of alcohol consumption on miR-122 liver expression^49^,^50^, but it seems certain that the development of ALD also affects miR-122 expression in the human liver^17^. Even modest amount of alcohol consumption has been show to elevate serum miR-122 levels^51^. Pathological states in the liver [NAFDL^18^,^28^, ALD^23^ cirrhosis^52^, cancer^29^, toxicity^53^ and infection^31^] have all been shown to be associated with altered serum/plasma levels of miR-122. In support to our results associating hsa-miR-122-5p levels with liver enzyme levels, the serum levels of miR-122 have been previously shown to be associated with ALT and total cholesterol in hepatitis C patients receiving a nucleic acid-based miR-122 inhibitor^54^. Priola *et al*. also showed significant correlation between miR-122 and ALT, AST and GT and their results indicate that miR-122 may even regulate ALT activity^34^.

The increased levels of hsa-miR-885-5p were associated with FL. Levels of this miRNA remained an independent predictor of FL when adjusting with age, sex, BMI and most of the risk factors of FL, including alcohol consumption, but the significance was abolished when insulin levels were added to predicting model. Previous studies have indicated that hsa-miR-885-5p expression is relatively liver tissue enriched^55^ but also peripheral blood mononuclear cells have been thought to express this miRNA^56^, indicating possible function in liver and in blood stream. In line with our results, up-regulation of plasma miR-885-5p levels has been reported in paracetamol induced liver toxicity^53^. In contrast to a previous finding of increased levels of miR-885-5p in liver cirrhosis and hepatocellular carcinoma in a small case control study^55^, we observed an independent and direct association between miR-885-5p and ALT, AST and GT concentrations in our wide population-based sample. In addition, we found for the first time that hsa-miR-885-5p levels were inversely correlated with large HDL cholesterol independently of age, sex, BMI, measured liver enzyme levels and liver fat status.

Blood hsa-miR-122-5p levels were comparable to serum liver enzyme levels when identifying individuals with FL. Adding hsa-miR-122-5p and -885-5p to a model containing traditional risk factors and biomarkers of FL improved the risk stratification of FL. Although the improvement was statistically significant, it was minor and, unlike in the study by Tan *et al*.^18^ or Pirola *et al*.^34^ in more select populations, our results do not support blood miRNA levels having considerable novel clinical value in the diagnostics of FL in general population with no clinical symptoms of FL.

Our results of the metabolomic analysis showed that hsa-miR-122-5p levels had a direct association with small VLDL, IDL, large LDL subfractions, and apoB levels, indicating that hsa-miR-122-5p is connected with cholesterol levels in a viral hepatitis-free human population. These results are well supported by animal studies, where knock-out, knock-down, and overexpression studies on rodents and non-human primates have indicated that miR-122 expression modifies serum cholesterol and triglyceride levels by controlling cholesterol synthesis and VLDL secretion, and that suppressing miR-122 expression lowers LDL levels and increases HDL levels^47^,^57^,^58^,^59^. Hsa-miR-122-5p has also been detected in HDL and LDL particles^14^, and thus transport in lipoproteins may contribute to the direct associations observed between hsa-miR-122-5p and lipoprotein subfraction levels. In the present study, hsa-miR-122-5p levels did not correlate with the leucocyte count, suggesting that the measured concentrations do originate from serum. We did not find any predicted target mRNAs, which would have correlated negatively and significantly (after multiple testing correction) with hsa-miR-122-5p levels, indicating that this miRNA functions mainly in the liver.

In our transcriptomics analyses, we observed a significant inverse correlation between hsa-miR-885-5p and its target OSBPL2 expression. The OSBPL protein family has been shown to act as either lipid transporters or sterol sensors that control lipid metabolism by being involved in reverse cholesterol transport, vesicle transport, and cell signaling and by suppressing hepatic lipogenesis and VLDL production^60^. OSBPL2 has also been previously shown affect the metabolism of neutral lipids, possibly integrating the cellular metabolism of triglycerides with that of cholesterol^61^. Furthermore, OSBPL2 overexpression has been shown to be associated with increased cell efflux of cholesterol, apoA1, and phosphatidyl choline vesicles to serum^62^. Suppressing the expression of OSBPL2 may be hypothesized to be the biological pathway through which elevated hsa-miR-885-5p is associated with decreased levels of large HDL cholesterol.

A limitation of our study is that profiling miRNAs from peripheral blood poses a challenge in identifying the origin of the analyzed miRNAs, as blood contains miRNAs from circulatory cells but also vesicle- and protein-bound miRNA originating from various tissues. Whole blood was selected to enable gene-expression analysis from the same sample. Although clinical diagnosis of ADL or NAFLD was not available, we had detailed information about patients lifetime alcohol consumption, liver enzyme levels, disease status and ultrasonic assessment of FL. This data was used to separate subjects with FL without significant alcohol use and FL with alcohol use. The detection of fatty liver relies on ultrasonic assessment performed as a part of a scientific follow-up study, and liver biopsy (histology) was not available due to ethical reasons. According to the Finnish Red Cross, the prevalence of hepatitis B and C are 0.03% and 0.05%, respectively, among Finnish blood donors and even though we have excluded individuals with ALT, AST and GT levels over the Nordic reference ranges from transcriptomics and metabolomics analysis, we cannot exclude that some of the study subject may have had steatohepatitis, which could affect the these results. In addition, majority of individuals with FL in our population are men. Even though sex has been included in statistical analyses as covariate and sex stratified analyses were performed, possible features specific for FL in women could have been missed due to low number of women with FL. The results cannot therefore be directly generalized to older populations with equal amounts of women and men with FL. The strengths of this study are the large, well-phenotyped population-based cohort and the availability of comprehensive molecular phenotyping by serum NMR metabolomics, which enables detailed analysis of specific biological processes through which miRNAs may exert their effects. As this work is essentially descriptive, more research is needed to shed light on the mechanism how miRNAs are released into the blood and to validate the interaction of the discovered miRNAs and the their targets, for example *In Vitro* studies confirming the connection between hsa-miR-885-5p and OSBPL2 are needed.

In summary, we found that hsa-miR-122-5p and -885-5p are significantly up-regulated in individuals with ultrasonographically defined FL in a large Finnish population-based study cohort (YFS). The blood levels of hsa-miR-122-5p and -885-5p slightly improved FL prediction beyond established risk factors. In the metabolomics analysis, hsa-miR-122-5p levels were associated directly with levels of small VLDL, IDL, and large LDL components and hsa-miR-885-5p levels were associated inversely with extra-large HDL particle lipids and cholesterol levels. Furthermore, we found significant and inverse association between hsa-miR-885-5p and OSBPL2, a protein known to affect efflux of cholesterol.

## Additional Information

**How to cite this article**: Raitoharju, E. *et al*. Blood hsa-miR-122-5p and hsa-miR-885-5p levels associate with fatty liver and related lipoprotein metabolism—The Young Finns Study. *Sci. Rep.* **6**, 38262; doi: 10.1038/srep38262 (2016).

**Publisher's note:** Springer Nature remains neutral with regard to jurisdictional claims in published maps and institutional affiliations.

## Supplementary Material

Supplementary Information

## Figures and Tables

**Table 1:** Demographics of the subjects of Young Finns Study population with successful miRNA profiling.

	All subjects	NL	FL_All_	FL_M_	FL_C_
Number of subjects	871	724	147	119	28
Age, years	42.4 (4.8)	42.1 (4.9)	43.6 (4.4)	43.5 (4.5)	44.3 (4.0)
Men, (%)	45.4	39.5	74.1	75.6	67.9
Total cholesterol, mmol/l	5.1 (0.9)	5.1 (0.9)	5.4 (1.0)	5.5 (1.0)	5.1 (1.1)
HDL cholesterol, mmol/l	1.3 (0.3)	1.4 (0.3)	1.2 (0.3)	1.2 (0.3)	1.1 (0.3)
LDL cholesterol, mmol/l	3.3 (0.8)	3.2 (0.8)	3.4 (0.9)	3.5 (1.0)	3.1 (0.9)
Triglycerides, mmol/l	1.2 (0.7)	1.1 (0.6)	1.8 (1.0)	1.8 (1.0)	1.8 (0.9)
Type 2 diabetes, (%)	1.7	1.2	10.9	9.2	17.9
Blood glucose, mmol/l	5.4 (0.9)	5.3 (0.8)	5.8 (1.0)	5.7 (1.0)	6.0 (1.1)
HbA1c, %	5.5 (0.4)	5.4 (0.4)	5.7 (0.6)	5.7 (0.5)	6.0 (0.7)
HbA1c, mmol/mol	36.5 (4.6)	36.0 (3.9)	39.1 (6.5)	38.5 (6.0)	41.8 (7.9)
Hypertension, (%)	8.2	5.4	21.8	17.6	39.3
Systolic BP, mmHg	119.1 (13.9)	117.3 (13.3)	127.7 (13.1)	127.0 (13.2)	130.8 (12.3)
Diastolic BP, mmHg	75.1 (10.5)	73.6 (9.9)	82.1 (10.4)	81.6 (10.8)	84.2 (8.2)
Body mass index, kg/m^2^	26.4 (4.9)	25.6 (4.3)	30.6 (5.8)	29.2 (4.0)	36.5 (8.2)
Alanine aminotransferase, U/l	16.9 (12.2)	14.3 (8.0)	29.6 (19.5)	28.0 (17.7)	36.4 (25.2)
Aspartate aminotransferase, U/l	22.2 (8.5)	21.0 (7.2)	28.2 (11.4)	27.4 (10.5)	31.3 (14.6)
γ-glutamyltransferase, U/l	30.6 (28.3)	25.6 (46.0)	55.6 (46.0)	54.8 (49.4)	58.8 (27.8)
Fatty liver index	3.8 (10.5)	1.9 (5.6)	13.3 (19.7)	8.5 (12.0)	33.3 (31.0)

Continuous variables are presented by means with standard deviations in parentheses.

**Abbreviations:** NL = normal liver, FL = fatty liver, FL_M = _mild fatty liver, FL_C_ = clearly indentified fatty liver, FL_All_ = all subjects with fatty liver, HDL = high-density lipoprotein, LDL = low-density lipoprotein, HbA1c = glycosylated hemoglobin, BP = blood pressure.

**Table 2:** Logistic regression models (1–3) predicting fatty liver (FL_All_ or FL_C_) with hsa-miR-122-5p or -885-5p and known risk factors and biomarkers of FL.

Outcome	MODEL 1		MODEL 2		MODEL 3	
	miR-122	miR-885	miR-122	miR-885	miR-122	miR-885
NL vs. FL_All_						
n	703	871	701	868	632	781
p-value	1.26 * 10^−14^	3.91 * 10^−6^	1.29 * 10^−8^	0.002	6.84 * 10^−5^	0.131
OR	2.44	1.55	2.07	1.41	1.78	1.23
95% CI	1.96–3.09	1.29–1.86	1.62–2.68	1.13–1.77	1.35–2.38	0.94–1.60
NL vs. FL_C_						
n	600	752	598	749	545	681
p-value	5.59 * 10^−9^	1.33 * 10^−4^	1.93 * 10^−6^	9.83 * 10^−4^	2.48 * 10^−3^	0.353
OR	4.61	2.14	6.67	2.47	3.64	1.37
95% CI	2.83–7.96	1.46–3.19	3.28–15.97	1.46–4.33	1.69–9.29	0.71–2.73

MODEL 1: *Stepwise logistic regression model predicting FL (either FL_All_ or FL_C_) with miR-122-5p or miR-885-5p (one by one forced into model).

MODEL 2: Model 1+ age, sex and BMI.

MODEL 3: Model 2+ alcohol consumption, waist circumference, apolipoprotein B levels, triglycerides, insulin levels, systolic blood pressure, smoking, and physical activity index. This model involves all the explanatory variables that have been previously associated with liver status in Young Finns Study [see ref. 6] excluding liver enzymes due to high correlation with studied miRNAs.

Abbreviations: NL = Normal liver, FL = fatty liver, FL_All_ = all subjects with fatty liver, FL_C_ = clearly identified fatty liver, miR-122 = hsa-miR-122-5p, miR-885 = hsa-miR-885-5p.

**Table 3:** Correlation and adjusted linear regressions between hsa-miR-122-5p and -885-5p and serum liver enzyme levels.

	Spearman correlation				Linear regression model^*^		
	n	p	p_c_	r	n	p	ß (95% CI)
Hsa-miR-122-5p							
ALT	703	5.01 * 10^−33^	1.22 * 10^−30^	0.43	701	1.07 * 10^−25^	0.319 (0.262–0.376)
AST	703	4.49 * 10^−26^	1.09 * 10^−23^	0.384	701	3.43 * 10^−22^	0.321 (0.258–0.384)
GT	703	5.43 * 10^−17^	1.32 * 10^−14^	0.309	701	2.10 * 10^−7^	0.163 (0.102–0.224)
Hsa-miR-885-5p							
ALT	868	3.78 * 10^−19^	9.18 * 10^−17^	0.297	868	9.33 * 10^−14^	0.377 (0.149–0.252)
AST	868	1.71 * 10^−16^	4.16 * 10^−14^	0.275	868	4.19 * 10^−12^	0.205 (0.148–0.262)
GT	868	1.26 * 10^−08^	3.05 * 10^−6^	0.192	868	4.97 * 10^−4^	0.096 (0.042–0.150)

^*^Statistical model: Stepwise linear regression model predicting liver enzyme levels (one by one) with miR-122-5p or miR-885-5p (one by one forced into model), age, sex, BMI, and liver status. Abbreviations: ALT = Alanine aminotransferase, AST = Aspartate aminotransferase, GT = gamma-glutamyltransferase, r = correlation estimate, p_c_ = Bonferroni corrected p-value. Betas (β) indicates the standard deviation (SD) change of the liver enzyme levels per increase of one SD of miRNA levels.

**Table 4:** Continuous net reclassification improvement (NRI) of risk stratification of fatty liver after adding hsa-miR-122-5p and hsa-885-5p to the different base models* including conventional risk factors.

Model/microRNAs	AUC	NRI (95% CI)	p-value
**Model 1** (**predicting FL**_**C**_**vs. NL**)	0.983	Reference	Reference
+hsa-miR-122-5p	0.986	0.963 (0.570–1.356)	1.6 * 10^−6^
+hsa-miR-885-5p	0.983	0.139 (−0.296–0.575)	0.53
+hsa-miR-122-5p and hsa-miR-885-5p	0.987	1.363 (1.057–1.670)	2.8 * 10^−18^
**Model 2** (**predicting FL**_**All**_ **vs. NL**)	0.897	Reference	Reference
+hsa-miR-122-5p	0.898	0.125 (−0.080–0.329)	0.23
+hsa-miR-885-5p	0.897	0.055 (−0.1515–0.262)	1.00
+hsa-miR-122-5p and hsa-miR-885-5p	0.898	0.278 (0.077–0.480)	0.0067

*Statistical model: Stepwise models 1 and 2 consist of all variables associated with FL in the Young Finns Study [see ref. 6].

Variables with independent and significant prediction value were: Model 1: Alanine aminotransferase (ALT), gamma-glutamyltransferase (GT), waist circumference, insulin levels predicting individuals with FL_C_ vs. NL (NL n = 524, FL_C_ n = 21).

Model 2: ALT, GT, waist circumference, insulin levels, and systolic blood pressure predicting individuals with FL_All_ vs NL (NL n = 524, FL_All_ n = 108).

Abbreviations: AUC = area under curve, NL = normal liver, FL = fatty liver, FL_All_ = all subjects with FL, FL_C_ = clearly identified fatty liver.

**Table 5:** Correlation and adjusted linear regressions between hsa-miR-122-5p and -885-5p and *in silico* predicted mRNA target expression levels from transcriptomics analysis.

Gene ID	Accession ID	Spearman correlation			Linear regression model*		
		n	p-value	r	n	p-value	β (CI(95%)
Hsa-miR-122-5p							
CS	NM_004077.2	616	0.002	0.122	586	0.005	0.131 (0.041–0.222)
ARSB	NM_000046.2	616	0.038	0.084	586	0.005	0.118 (0.036–0.201)
TRAK1	NM_014965.2	616	0.002	0.123	586	0.009	0.122 (0.031–0.213)
PTP4A1	NM_003463.3	616	0.004	0.114	586	0.011	0.113 (0.026–0.200)
CCDC69	NM_015621.2	616	0.032	0.087	586	0.013	0.106 (0.023–0.190)
TRAK1	NM_001042646.1	616	0.032	0.086	586	0.013	0.109 (0.023–0.194)
CCDC43	NM_144609.1	616	0.032	0.086	586	0.014	0.102 (0.021–0.182)
BCL2L2	NM_004050.2	616	0.029	0.088	586	0.014	0.114 (0.023–0.205)
LCMT1	NM_016309.2	616	0.033	0.086	586	0.014	0.105 (0.021–0.188)
EIF1AX	NM_001412.3	616	0.014	−0.099	586	0.016	−0.111 (−0.201–−0.021)
LAMC1	NM_002293.2	616	0.016	0.097	586	0.018	0.103 (0.018–0.188)
SHCBP1	NM_024745.2	616	0.047	0.080	586	0.020	0.105 (0.017–0.194)
SORT1	NM_002959.4	616	0.020	0.094	586	0.031	0.099 (0.009–0.189)
CANX	NM_001024649.1	616	0.003	0.118	586	0.037	0.097 (0.006–0.188)
ZC3H10	NM_032786.1	616	0.033	−0.086	586	0.039	−0.090 (−0.175–−0.005)
PPAPDC1B	NM_032483.2	616	0.044	0.081	586	0.041	0.096 (0.004–0.187)
GALNT10	NM_198321.2	616	0.042	0.082	586	0.042	0.097 (0.004–0.191)
SUSD1	NM_022486.3	616	0.001	0.138	586	0.042	0.085 (0.003–0.167)
ATP5A1	NM_004046.4	616	0.044	0.081	586	0.049	0.092 (0.001–0.184)
Hsa-miR-885-5p							
GABARAP	NM_007278.1	740	0.032	−0.079	712	0.002	−0.120 (−0.194–−0.046)
ARSA	NM_000487.3	740	0.022	−0.084	712	0.005	−0.111 (−0.188–−0.035)
AHNAK	NM_024060.2	740	3.2 * 10^−5^	0.152	712	0.013	0.094 (0.020–0.168)
RSPH3	NM_031924.3	740	0.002	−0.111	712	0.014	−0.094 (−0.169–−0.019)
OSBPL2	NM_144498.1	740	1.0 * 10^−4^	−0.143	712	0.015	−0.095 (−0.171–−0.019)
UQCC	NM_199487.1	740	0.001	0.126	712	0.015	0.095 (0.019–0.172)
HNRNPL	NM_001005335.1	740	0.032	0.079	712	0.018	0.091 (0.016–0.166)
TRO	NM_001039705.1	740	4.6 * 10^−5^	0.149	712	0.019	0.092 (0.015–0.169)
FCRL6	NM_001004310.1	740	0.041	0.075	712	0.022	0.093 (0.014–0.172)
APOL3	NM_030644.1	740	0.001	0.118	712	0.022	0.088 (0.013–0.164)
PPP2R1A	NM_014225.3	740	0.005	0.102	712	0.028	0.087 (0.010–0.165)

*Statistical model: *In silico* predicted gene target (gene ID) expression predicted with FL associated miRNAs (one by one forced in the model) adjusted with age, sex, BMI, liver status, alanine aminotransferase (ALT), aspartate aminotransferase (AST) and gamma-glutamyltransferase (GT). Note: Individuals with ALT, AST or GT levels over the Finnish reference ranges were removed from the analysis.

## References

[b1] Angulo P. Nonalcoholic fatty liver disease. N. Engl. J. Med. 346, 1221–1231 (2002). 11961152 10.1056/NEJMra011775

[b2] Neuschwander-Tetri B. A. & Caldwell S. H. Nonalcoholic steatohepatitis: summary of an AASLD Single Topic Conference. Hepatology 37, 1202–1219 (2003). 12717402 10.1053/jhep.2003.50193

[b3] Sookoian S. & Pirola C. J. Systems biology elucidates common pathogenic mechanisms between nonalcoholic and alcoholic-fatty liver disease. PLoS One 8, e58895 (2013). 23516571 10.1371/journal.pone.0058895 PMC3596348

[b4] Targher G., Day C. P. & Bonora E. Risk of cardiovascular disease in patients with nonalcoholic fatty liver disease. N. Engl. J. Med. 363, 1341–1350 (2010). 20879883 10.1056/NEJMra0912063

[b5] Kotronen A. . Non-alcoholic and alcoholic fatty liver disease - two diseases of affluence associated with the metabolic syndrome and type 2 diabetes: the FIN-D2D survey. BMC Public Health 10, 237-2458-10-237 (2010). 10.1186/1471-2458-10-237 PMC2873937 20459722

[b6] Suomela E. . Prevalence and determinants of fatty liver in normal-weight and overweight young adults. The Cardiovascular Risk in Young Finns Study. Ann. Med. 47, 1–7 (2014). 25333756 10.3109/07853890.2014.966752

[b7] Cheng H. Y. . Nonalcoholic Fatty Liver Disease: Prevalence, Influence on Age and Sex, and Relationship with Metabolic Syndrome and Insulin Resistance. Int J Gerontol. 7, 194–198 (2013).

[b8] Kestila P. . Socioeconomic status, cardiovascular risk factors, and subclinical atherosclerosis in young adults: the cardiovascular risk in Young Finns Study. Arterioscler. Thromb. Vasc. Biol. 32, 815–821 (2012). 22223734 10.1161/ATVBAHA.111.241182

[b9] Silvestri P. . MicroRNAs and ischemic heart disease: towards a better comprehension of pathogenesis, new diagnostic tools and new therapeutic targets. Recent patents on cardiovascular drug discovery 4, 109–18 (2009). 19519553 10.2174/157489009788452977

[b10] Bartel D. P. MicroRNAs: target recognition and regulatory functions. Cell 136, 215–233 (2009). 19167326 10.1016/j.cell.2009.01.002 PMC3794896

[b11] Wang K., Zhang S., Weber J., Baxter D. & Galas D. J. Export of microRNAs and microRNA-protective protein by mammalian cells. Nucleic Acids Res. 38, 7248–7259 (2010). 20615901 10.1093/nar/gkq601 PMC2978372

[b12] Hoefer I. E. . Novel methodologies for biomarker discovery in atherosclerosis. Eur. Heart J. 36, 2635–2642 (2015). 26049157 10.1093/eurheartj/ehv236

[b13] Kosaka N. . Secretory mechanisms and intercellular transfer of microRNAs in living cells. J. Biol. Chem. 285, 17442–17452 (2010). 20353945 10.1074/jbc.M110.107821 PMC2878508

[b14] Vickers K. C., Palmisano B. T., Shoucri B. M., Shamburek R. D. & Remaley A. T. MicroRNAs are transported in plasma and delivered to recipient cells by high-density lipoproteins. Nat. Cell Biol. 13, 423–433 (2011). 21423178 10.1038/ncb2210 PMC3074610

[b15] Valadi H. . Exosome-mediated transfer of mRNAs and microRNAs is a novel mechanism of genetic exchange between cells. Nat. Cell Biol. 9, 654–659 (2007). 17486113 10.1038/ncb1596

[b16] Cheung O. . Nonalcoholic steatohepatitis is associated with altered hepatic MicroRNA expression. Hepatology 48, 1810–1820 (2008). 19030170 10.1002/hep.22569 PMC2717729

[b17] Liu Y., Chen S. H., Jin X. & Li Y. M. Analysis of differentially expressed genes and microRNAs in alcoholic liver disease. Int. J. Mol. Med. 31, 547–554 (2013). 23337955 10.3892/ijmm.2013.1243

[b18] Tan Y., Ge G., Pan T., Wen D. & Gan J. A pilot study of serum microRNAs panel as potential biomarkers for diagnosis of nonalcoholic fatty liver disease. PLoS One 9, e105192 (2014). 25141008 10.1371/journal.pone.0105192 PMC4139327

[b19] Chen Y. P., Jin X., Xiang Z., Chen S. H. & Li Y. M. Circulating MicroRNAs as potential biomarkers for alcoholic steatohepatitis. Liver Int. 33, 1257–1265 (2013). 23682678 10.1111/liv.12196

[b20] Cermelli S., Ruggieri A., Marrero J. A., Ioannou G. N. & Beretta L. Circulating microRNAs in patients with chronic hepatitis C and non-alcoholic fatty liver disease. PLoS One 6, e23937 (2011). 21886843 10.1371/journal.pone.0023937 PMC3160337

[b21] Murakami Y. . Comprehensive miRNA expression analysis in peripheral blood can diagnose liver disease. PLoS One 7, e48366 (2012). 23152743 10.1371/journal.pone.0048366 PMC3485241

[b22] Fernandez-Hernando C., Ramirez C. M., Goedeke L. & Suarez Y. MicroRNAs in metabolic disease. Arterioscler. Thromb. Vasc. Biol. 33, 178–185 (2013). 23325474 10.1161/ATVBAHA.112.300144 PMC3740757

[b23] Bala S. . Circulating microRNAs in exosomes indicate hepatocyte injury and inflammation in alcoholic, drug-induced, and inflammatory liver diseases. Hepatology 56, 194–1957 (2012). 10.1002/hep.25873 PMC3486954 22684891

[b24] Murakami Y. . The progression of liver fibrosis is related with overexpression of the miR-199 and 200 families. PLoS One 6, e16081 (2011). 21283674 10.1371/journal.pone.0016081 PMC3025920

[b25] Murakami Y. . Comprehensive analysis of microRNA expression patterns in hepatocellular carcinoma and non-tumorous tissues. Oncogene 25, 2537–2545 (2006). 16331254 10.1038/sj.onc.1209283

[b26] Byrne C. D. & Targher G. NAFLD: a multisystem disease. J. Hepatol. 62, S47–64 (2015). 25920090 10.1016/j.jhep.2014.12.012

[b27] Altamirano J. & Bataller R. Alcoholic liver disease: pathogenesis and new targets for therapy. Nat. Rev. Gastroenterol. Hepatol. 8, 491–501 (2011). 21826088 10.1038/nrgastro.2011.134

[b28] Yamada H. . Associations between circulating microRNAs (miR-21, miR-34a, miR-122 and miR-451) and non-alcoholic fatty liver. Clin. Chim. Acta 424, 99–103 (2013). 23727030 10.1016/j.cca.2013.05.021

[b29] Koberle V. . Serum microRNA-1 and microRNA-122 are prognostic markers in patients with hepatocellular carcinoma. Eur. J. Cancer 49, 3442–3449 (2013). 23810247 10.1016/j.ejca.2013.06.002

[b30] Celikbilek M. . Circulating microRNAs in patients with non-alcoholic fatty liver disease. World J. Hepatol. 6, 613–620 (2014). 25232454 10.4254/wjh.v6.i8.613 PMC4163744

[b31] Akamatsu S. . Differences in serum microRNA profiles in hepatitis B and C virus infection. J. Infect. 70, 273–287 (2015). 25452043 10.1016/j.jinf.2014.10.017

[b32] Szabo G. & Bala S. MicroRNAs in liver disease. Nat. Rev. Gastroenterol. Hepatol. 10, 542–552 (2013). 23689081 10.1038/nrgastro.2013.87 PMC4091636

[b33] Gori M., Arciello M. & Balsano C. MicroRNAs in nonalcoholic fatty liver disease: novel biomarkers and prognostic tools during the transition from steatosis to hepatocarcinoma. Biomed. Res. Int. 2014, 741465 (2014). 24745023 10.1155/2014/741465 PMC3972908

[b34] Pirola C. J. . Circulating microRNA signature in non-alcoholic fatty liver disease: from serum non-coding RNAs to liver histology and disease pathogenesis. Gut 64, 800–812 (2015). 24973316 10.1136/gutjnl-2014-306996 PMC4277726

[b35] Raitakari O. T. . Cohort profile: the cardiovascular risk in Young Finns Study. International journal of epidemiology 37, 1220–6 (2008). 18263651 10.1093/ije/dym225

[b36] Soininen P. . High-throughput serum NMR metabonomics for cost-effective holistic studies on systemic metabolism. Analyst 134, 1781–1785 (2009). 19684899 10.1039/b910205a

[b37] Soininen P., Kangas A. J., Würtz P., Suna T. & Ala-Korpela M. Quantitative serum nuclear magnetic resonance metabolomics in cardiovascular epidemiology and genetics. Circ. Cardiovasc. Genet. 8, 192–206 (2015). 25691689 10.1161/CIRCGENETICS.114.000216

[b38] Inouye M. . Metabonomic, transcriptomic, and genomic variation of a population cohort. Mol. Syst. Biol. 6, 441 (2010). 21179014 10.1038/msb.2010.93 PMC3018170

[b39] Edens M. A. . Ultrasonography to quantify hepatic fat content: validation by 1H magnetic resonance spectroscopy. Obesity (Silver Spring) 17, 2239–2244 (2009). 19461588 10.1038/oby.2009.154

[b40] Saverymuttu S. H., Joseph A. E. & Maxwell J. D. Ultrasound scanning in the detection of hepatic fibrosis and steatosis. Br. Med. J. (Clin. Res. Ed) 292, 13–15 (1986). 10.1136/bmj.292.6512.13 PMC1338970 3080046

[b41] Leidinger P., Backes C., Meder B., Meese E. & Keller A. The human miRNA repertoire of different blood compounds. BMC Genomics 15, 474-2164-15-474 (2014). 10.1186/1471-2164-15-474 PMC4076980 24928098

[b42] Raitoharju E. . Blood microRNA profile associates with the levels of serum lipids and metabolites associated with glucose metabolism and insulin resistance and pinpoints pathways underlying metabolic syndrome: The cardiovascular risk in Young Finns Study. Mol. Cell. Endocrinol. 391, 41–49 (2014). 24784704 10.1016/j.mce.2014.04.013

[b43] Elovainio M. . Activated immune-inflammatory pathways are associated with long-standing depressive symptoms: Evidence from gene-set enrichment analyses in the Young Finns Study. J. Psychiatr. Res. 71, 120–125 (2015). 26473696 10.1016/j.jpsychires.2015.09.017

[b44] Bedogni G. . The Fatty Liver Index: a simple and accurate predictor of hepatic steatosis in the general population. BMC Gastroenterol. 6, 33 (2006). 17081293 10.1186/1471-230X-6-33 PMC1636651

[b45] Cho S. . MiRGator v3.0: a microRNA portal for deep sequencing, expression profiling and mRNA targeting. Nucleic Acids Res. 41, D252–7 (2013). 23193297 10.1093/nar/gks1168 PMC3531224

[b46] Chang J. . miR-122, a mammalian liver-specific microRNA, is processed from hcr mRNA and may downregulate the high affinity cationic amino acid transporter CAT-1. RNA Biol. 1, 106–113 (2004). 17179747 10.4161/rna.1.2.1066

[b47] Hsu S. H. . Essential metabolic, anti-inflammatory, and anti-tumorigenic functions of miR-122 in liver. J. Clin. Invest. 122, 2871–2883 (2012). 22820288 10.1172/JCI63539 PMC3408748

[b48] Tsai W. C. . MicroRNA-122 plays a critical role in liver homeostasis and hepatocarcinogenesis. J. Clin. Invest. 122, 2884–2897 (2012). 22820290 10.1172/JCI63455 PMC3408747

[b49] McDaniel K. . The functional role of microRNAs in alcoholic liver injury. J. Cell. Mol. Med. 18, 197–207 (2014). 24400890 10.1111/jcmm.12223 PMC3930407

[b50] Dippold R. P., Vadigepalli R., Gonye G. E., Patra B. & Hoek J. B. Chronic ethanol feeding alters miRNA expression dynamics during liver regeneration. Alcohol. Clin. Exp. Res. 37 Suppl 1, E59–69 (2013). 22823254 10.1111/j.1530-0277.2012.01852.x PMC3482264

[b51] McCrae J. C., Sharkey N., Webb D. J., Vliegenthart A. D. & Dear J. W. Ethanol consumption produces a small increase in circulating miR-122 in healthy individuals. Clin. Toxicol. (Phila) 54, 53–55 (2016). 26574140 10.3109/15563650.2015.1112015

[b52] Sohn W. . Serum exosomal microRNAs as novel biomarkers for hepatocellular carcinoma. Exp. Mol. Med. 47, e184 (2015). 26380927 10.1038/emm.2015.68 PMC4650928

[b53] Vliegenthart A. D. . Comprehensive microRNA profiling in acetaminophen toxicity identifies novel circulating biomarkers for human liver and kidney injury. Sci. Rep. 5, 15501 (2015). 26489516 10.1038/srep15501 PMC4614545

[b54] Janssen H. L. . Treatment of HCV infection by targeting microRNA. N. Engl. J. Med. 368, 1685–1694 (2013). 23534542 10.1056/NEJMoa1209026

[b55] Gui J. . Serum microRNA characterization identifies miR-885-5p as a potential marker for detecting liver pathologies. Clin. Sci. (Lond) 120, 183–193 (2011). 20815808 10.1042/CS20100297 PMC2990200

[b56] Guan X. . A functional variant at the miR-885-5p binding site of CASP3 confers risk of both index and second primary malignancies in patients with head and neck cancer. FASEB J. 27, 1404–1412 (2013). 23271051 10.1096/fj.12-223420 PMC3606531

[b57] Elmen J. . Antagonism of microRNA-122 in mice by systemically administered LNA-antimiR leads to up-regulation of a large set of predicted target mRNAs in the liver. Nucleic Acids Res. 36, 1153–1162 (2008). 18158304 10.1093/nar/gkm1113 PMC2275095

[b58] Elmen J. . LNA-mediated microRNA silencing in non-human primates. Nature 452, 896–899 (2008). 18368051 10.1038/nature06783

[b59] Esau C. . miR-122 regulation of lipid metabolism revealed by *in vivo* antisense targeting. Cell. Metab. 3, 87–98 (2006). 16459310 10.1016/j.cmet.2006.01.005

[b60] Zhou Y. . OSBP-related proteins (ORPs) in human adipose depots and cultured adipocytes: evidence for impacts on the adipocyte phenotype. PLoS One 7, e45352 (2012). 23028956 10.1371/journal.pone.0045352 PMC3448648

[b61] Hynynen R. . OSBP-related protein 2 is a sterol receptor on lipid droplets that regulates the metabolism of neutral lipids. J. Lipid Res. 50, 1305–1315 (2009). 19224871 10.1194/jlr.M800661-JLR200 PMC2694330

[b62] Laitinen S. . ORP2, a homolog of oxysterol binding protein, regulates cellular cholesterol metabolism. J. Lipid Res. 43, 245–255 (2002). 11861666

