# Supplementary material for: Blood hsa-miR-122-5p and hsa-miR-885-5p levels associate with fatty liver and related lipoprotein metabolism—The Young Finns Study
Source: Sci Rep. 2016 Dec 5;6:38262. doi: 10.1038/srep38262 (PMC5137183; doi:10.1038/srep38262)
Supplement: Supplementary Information [file srep38262-s1.pdf]

Supplementary material

Blood hsa-miR-122-5p and hsa-miR-885-5p levels associate with fatty liver and related lipoprotein metabolism¾ The Young Finns Study

Emma Raitoharju, Ilkka Seppälä, Leo-Pekka Lyytikäinen, Jorma Viikari, Mika Ala-Korpela, Pasi Soininen, Antti J. Kangas, Melanie Waldenberger, Norman Klopp, Thomas Illig, Jaana Leiviskä, Britt-Marie Loo, Niku Oksala, Mika Kähönen, Nina Hutri-Kähönen, Reijo Laaksonen, Olli Raitakari, Terho Lehtimäki

Table of contents

Supplementary figures 2
- Supplementary Figure 1. 2
- Supplementary Figure 2. 3

Supplementary tables 4
- Supplementary Table 1. 4
- Supplementary Table 2. 8
- Supplementary Table 3. 9
- Supplementary Table 4. 10
- Supplementary Table 5. 12
- Supplementary Table 6. 13

Supplementary figures

Supplementary Figure 1. Flow and summary of the study.

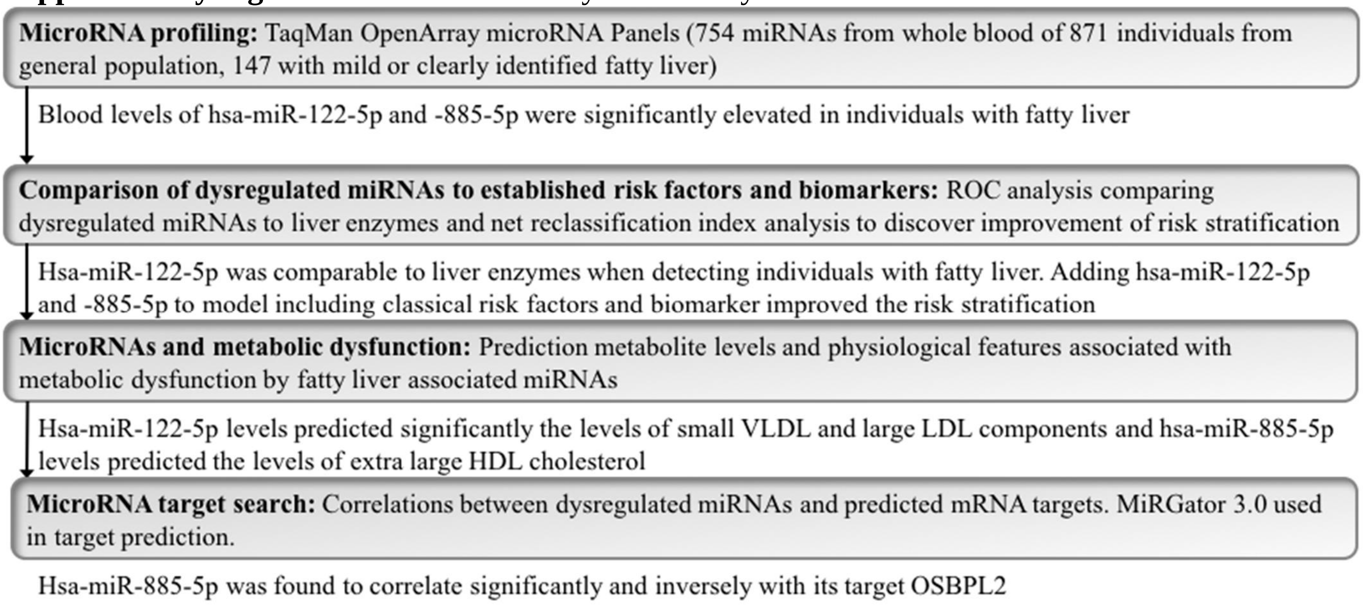

Supplementary Figure 2. Blood levels of hsa-miR-122-5p (A) and hsa-miR-885-5p (B) in individuals without fatty liver (NL), or with mild (FL_M) or clearly indentified fatty liver (FL_C) separately for those with and without excess alcohol cosumption (>20g of ethanol per day) to represent alcoholic and non-alcoholic fatty liver.

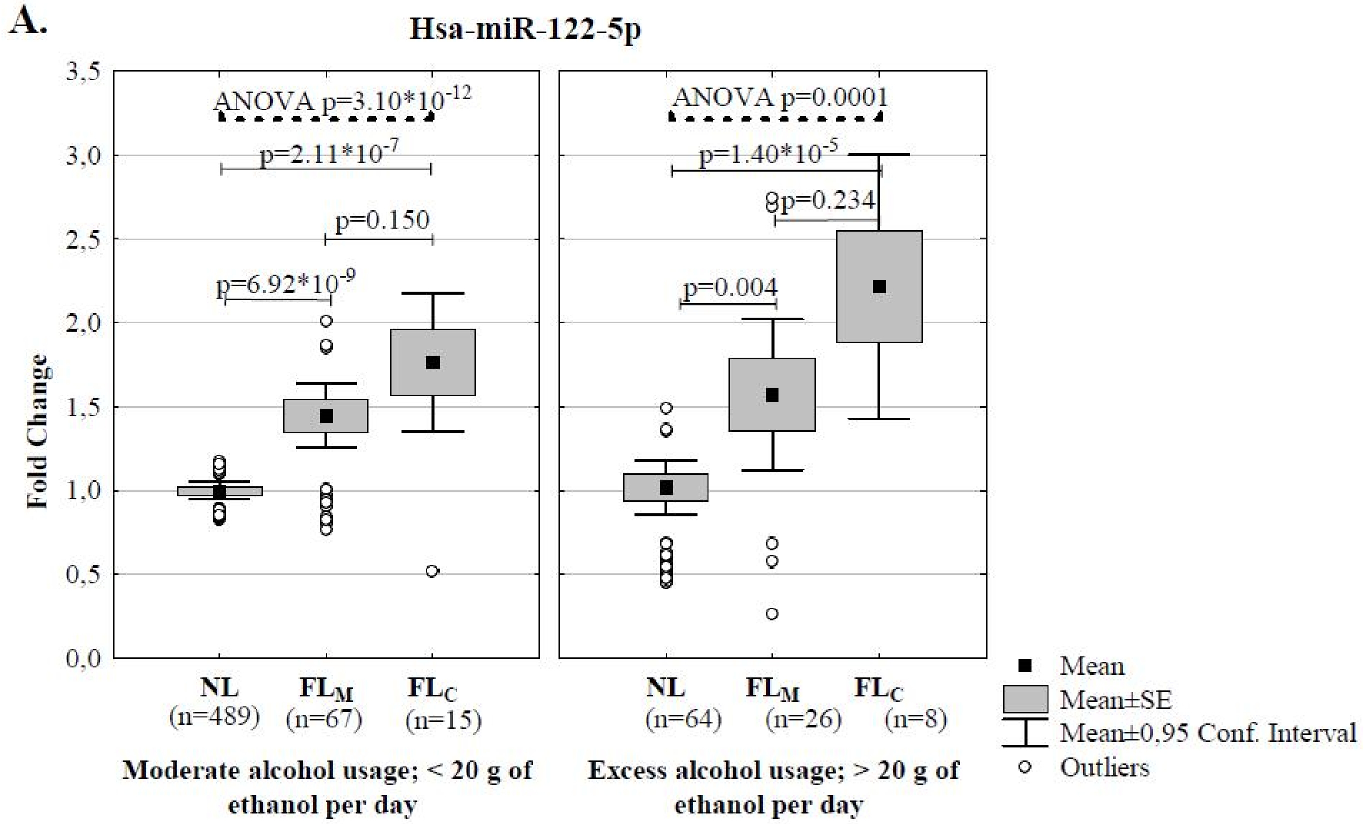

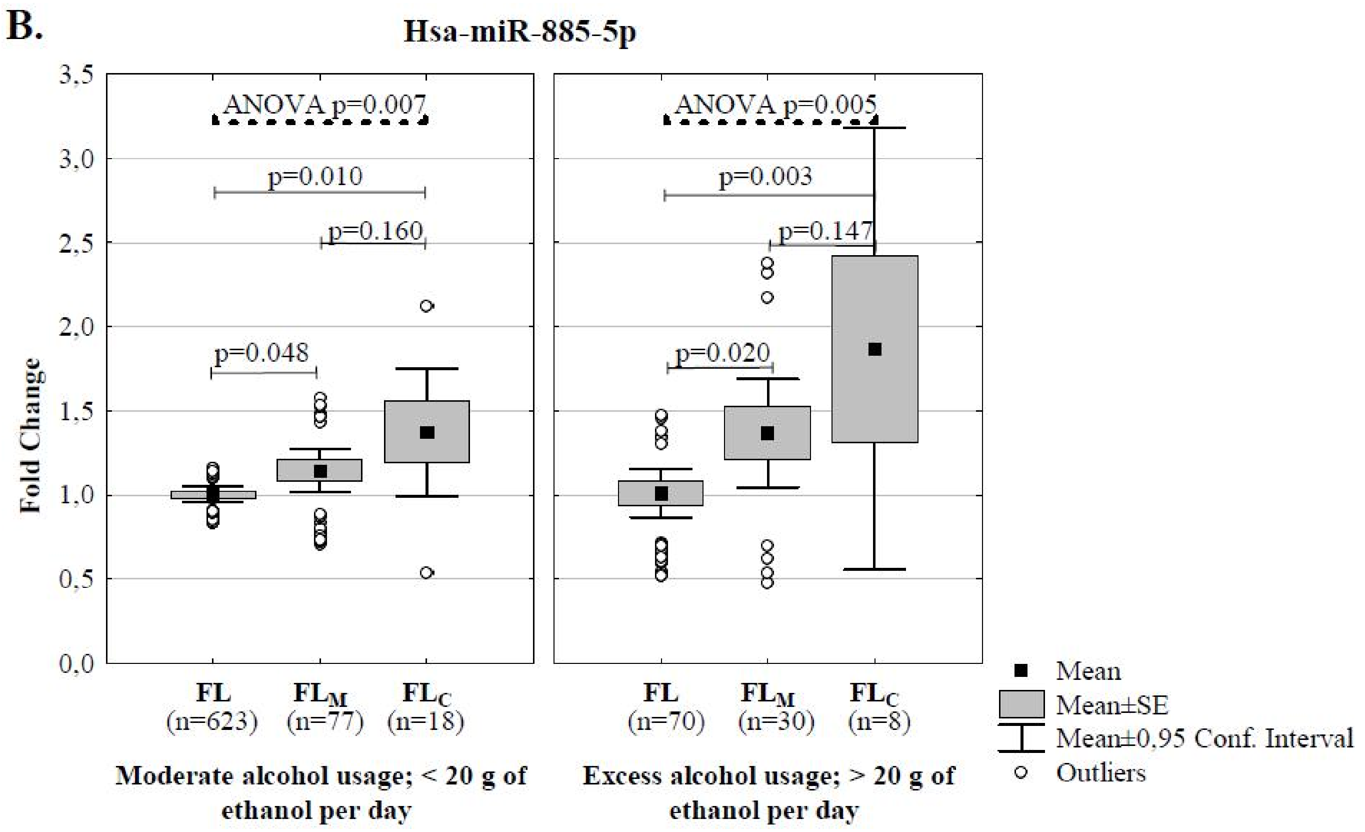

Abbreviations: ANOVA = Analysis of variance, SE = standard error.

Supplementary tables

Supplementary Table 1. List of metabolites and conventional biochemical and anthropometric measurements correlated with miRNA expression.

Metabolite/measurement	Unit	Mean	Standard deviation	Analysis Method
**Lipoprotein particle concentrations**				
Extremely large VLDL	µmol/L	$7.38*10^{-5}$	$1.07*10^{-4}$	NMR
Very large VLDL	µmol/L	$4.14*10^{-4}$	$6.64*10^{-4}$	NMR
Large VLDL	µmol/L	$3.8*10^{-4}$	$4.35*10^{-3}$	NMR
Medium VLDL	µmol/L	0.02	0.01	NMR
Small VLDL	µmol/L	0.03	0.01	NMR
Very small VLDL	µmol/L	0.04	0.01	NMR
IDL	µmol/L	0.10	0.02	NMR
Large LDL	µmol/L	0.17	0.04	NMR
Medium LDL	µmol/L	0.14	0.04	NMR
Small LDL	µmol/L	0.15	0.04	NMR
Very large HDL	µmol/L	0.32	0.21	NMR
Large HDL	µmol/L	1.11	0.51	NMR
Medium HDL	µmol/L	1.98	0.37	NMR
Small HDL	µmol/L	4.66	0.45	NMR
**Lipoprotein lipid concentrations**				
Extremely large VLDL	mmol/L	0.01	0.02	NMR
Very large VLDL	mmol/L	0.04	0.07	NMR
Large VLDL	mmol/L	0.22	0.26	NMR
Medium VLDL	mmol/L	0.55	0.40	NMR
Small VLDL	mmol/L	0.61	0.27	NMR
Very small VLDL	mmol/L	0.52	0.15	NMR
IDL	mmol/L	1.22	0.30	NMR
Large LDL	mmol/L	1.53	0.39	NMR
Medium LDL	mmol/L	0.91	0.24	NMR
Small LDL	mmol/L	0.56	0.16	NMR
Very large HDL	mmol/L	0.37	0.24	NMR
Large HDL	mmol/L	0.84	0.41	NMR
Medium HDL	mmol/L	1.06	0.20	NMR
Small HDL	mmol/L	1.27	0.14	NMR
**Lipoprotein phospholipid concentrations**				
Total	mmol/L	0.85	0.20	NMR
Extremely large VLDL	mmol/L	0.001	0.002	NMR
Very large VLDL	mmol/L	0.01	0.01	NMR
Large VLDL	mmol/L	0.04	0.05	NMR
Medium VLDL	mmol/L	0.11	0.07	NMR
Small VLDL	mmol/L	0.14	0.06	NMR
Very small VLDL	mmol/L	0.16	0.05	NMR
IDL	mmol/L	0.34	0.08	NMR
Large LDL	mmol/L	0.37	0.08	NMR
Medium LDL	mmol/L	0.23	0.05	NMR
Very large HDL	mmol/L	0.20	0.13	NMR
Large HDL	mmol/L	0.43	0.18	NMR
Medium HDL	mmol/L	0.49	0.09	NMR
**Cholesterol**				
Total	mmol/L	5.15	0.91	Biochemical
Total	mmol/L	5.04	0.97	NMR
Large VLDL	mmol/L	0.04	0.05	NMR

Medium VLDL	mmol/L	0.15	0.09	NMR
Small VLDL	mmol/L	0.23	0.09	NMR
IDL	mmol/L	0.75	0.19	NMR
IDL	mmol/L	0.23	0.10	Calculated
LDL	mmol/L	3.07	0.70	Calculated
Large LDL	mmol/L	1.03	0.28	NMR
Medium LDL	mmol/L	0.61	0.18	NMR
Small LDL	mmol/L	0.36	0.11	NMR
Very large HDL	mmol/L	0.16	0.11	NMR
Large HDL	mmol/L	0.38	0.22	NMR
Medium HDL	mmol/L	0.52	0.11	NMR
HDL	mmol/L	1.3	0.3	Biochemical
HDL	mmol/L	1.63	0.39	NMR
HDL2	mmol/L	1.10	0.39	NMR
HDL3	mmol/L	0.53	0.04	NMR
LDL	mmol/L	2.00	0.57	NMR
**Cholesterol esters**				
Total	mmol/L	3.63	0.71	NMR
Large VLDL	mmol/L	0.02	0.03	NMR
Medium VLDL	mmol/L	0.08	0.05	NMR
Large LDL	mmol/L	0.75	0.21	NMR
Medium LDL	mmol/L	0.45	0.14	NMR
Very large HDL	mmol/L	0.11	0.08	NMR
Large HDL	mmol/L	0.30	0.17	NMR
Medium HDL	mmol/L	0.42	0.09	NMR
**Free cholesterol**				
Total	mmol/L	1.40	0.28	NMR
Large VLDL	mmol/L	0.02	0.03	NMR
Medium VLDL	mmol/L	0.06	0.05	NMR
Small VLDL	mmol/L	0.09	0.04	NMR
IDL	mmol/L	0.23	0.06	NMR
Large LDL	mmol/L	0.29	0.07	NMR
Very large HDL	mmol/L	0.05	0.03	NMR
Large HDL	mmol/L	0.08	0.05	NMR
Medium HDL	mmol/L	0.10	0.02	NMR
**Triglycerides**				
Total	mmol/L	1.22	0.72	Biochemical
Total	mmol/L	1.29	0.64	NMR
VLDL	mmol/L	0.80	0.57	NMR
VLDL	mmol/L	0.70	0.49	Calculated
Extremely large VLDL	mmol/L	0.01	0.02	NMR
Very large VLDL	mmol/L	0.03	0.04	NMR
Large VLDL	mmol/L	0.14	0.16	NMR
Medium VLDL	mmol/L	0.29	0.23	NMR
Small VLDL	mmol/L	0.24	0.13	NMR
Very small VLDL	mmol/L	0.12	0.04	NMR
IDL	mmol/L	0.14	0.04	NMR
Very large HDL	mmol/L	0.01	0.01	NMR
Small HDL	mmol/L	0.05	0.02	NMR
**Lipoprotein particle size**				
VLDL	nm	36.3	1.46	NMR
LDL	nm	23.6	0.16	NMR
HDL	nm	9.91	0.27	NMR

**Apolipoproteins**				
Apolipoprotein A1	g/L	1.60	0.24	Biochemical
Apolipoprotein A1	g/L	1.70	0.24	Estimated
Apolipoprotein B	g/L	1.04	0.27	Biochemical
Apolipoprotein B	g/L	0.95	0.24	Estimated
Apolipoprotein B/ Apolipoprotein A1		0.57	0.15	Calculated
Lipoprotein (a)	mg/L	164.7	171.0	Biochemical
**Glycolysis-related metabolites and hormones**				
Citrate	µmol/L	0.10	0.02	NMR
GHba1c	mmol/mol	36.5	4.58	Biochemical
GHba1c %	%	5.49	0.42	Calculated
Glucose	mmol/L	4.71	0.75	NMR
Glucose	mmol/L	5.35	0.87	Biochemical
Glycerol	µmol/L	0.10	0.04	NMR
Insulin	mU/l	9.87	15.4	Biochemical
Lactate	mmol/L	1.46	037	NMR
Pyruvate	µmol/L	0.08	0.02	NMR
**Fatty acids**				
Total fatty acids	mmol/L	11.0	2.65	NMR
Omega-3 fatty acids	mmol/L	0.42	0.14	NMR
Omega-6 fatty acids	mmol/L	3.68	0.69	NMR
Omega-9 and saturated fatty acids	mmol/L	6.92	2.03	NMR
Linoleic acid	mmol/L	3.10	0.60	NMR
Other polyunsaturated fatty acids than linoleic acid	mmol/L	2.14	0.57	NMR
Docosahexaenoic acid	mmol/L	0.17	0.07	NMR
Monounsaturated fatty acids	mmol/L	3.17	1.08	NMR
Omega-3 fatty acids per total fatty acids		3.86	1.05	Calculated
Omega-6 fatty acids per total fatty acids		33.9	3.89	Calculated
Omega-9 and saturated fatty acids per total fatty acids		62.2	4.01	Calculated
**Quality of lipids and fatty acids**				
Methylene groups per fatty acid		9.68	0.26	NMR
Triglycerides per Phosphoglycerides		1.21	0.56	NMR
Methylene groups per double bond		7.72	0.64	NMR
Double bonds per Fatty Acids		1.26	0.08	NMR
Bisallylic groups per double bond		0.54	0.03	NMR
Bisallylic groups per total fatty acids		0.68	0.08	NMR
Average fatty acid chain length		18.0	0.24	NMR
Double bond protons of mobile lipids	mmol/L	1.71	0.52	NMR
Methylene groups of mobile lipids	mmol/L	26.3	12.8	NMR
Methyl groups of mobile lipids	mmol/L	9.30	2.35	NMR
Phosphatidylcholine and other cholines	mmol/L	2.00	0.41	NMR
Sphingomyelins	mmol/L	0.26	0.05	NMR
**Amino Acids**				
Alanine	µmol/L	0.40	0.06	NMR
Glutamine	µmol/L	0.60	0.07	NMR
Glycine	µmol/L	0.29	0.06	NMR
Histidine	µmol/L	0.07	0.01	NMR
Isoleucine	µmol/L	0.05	0.02	NMR
Leucine	µmol/L	0.08	0.02	NMR

Phenylalanine	µmol/L	0.07	0.01	NMR
Tyrosine	µmol/L	0.05	0.01	NMR
Valine	µmol/L	0.20	0.04	NMR
**Ketones**				
3-hydroxybutyrate	mmol/L	0.10	0.11	NMR
Acetate	mmol/L	0.05	0.01	NMR
Acetoacetate	mmol/L	0.06	0.04	NMR
**Liver enzymes**				
Alanine aminotransferase	U/L	16.9	12.2	Biochemical
Aspartate aminotransferase	U/L	22.2	8.51	Biochemical
Gamma-glutamyltransferase	U/L	30.6	28.3	Biochemical
**Kidney function**				
Creatinine	mmol/L	0.06	0.01	NMR
Creatinine	mmol/L	0.08	0.01	Biochemical
**Inflammatory markers**				
C-reactive protein	mg/L	1.56	2.51	Biochemical
Alpha-1 glycoprotein	mmol/L	1.54	0.22	NMR
**Blood pressure**				
Systolic blood pressure	mmHg	119.1	13.9	Sphygmomanometer
Diastolic blood pressure	mmHg	75.1	10.5	Sphygmomanometer
**Anthropometric measurements**				
Waist circumference	cm	91.3	13.8	Measuring tape
Hip circumference	cm	101.6	9.0	Measuring tape
Waist-to-hip ratio	-	0.90	0.09	Calculated
Weight	kg	78.8	17.1	Scale
Height	cm	172.4	9.11	Measuring tape
Body mass index	kg/m^2	26.4	4.93	Calculated
**Blood count**				
Erythrocytes	x^{12}/L	4.67	0.42	Flow cytometry
Leukocytes	$x^9/$ L	5.49	1.45	Flow cytometry
Thrombocytes	x^{10} L	356.3	58.1	Flow cytometry
Hemoglobin	g/L	141.4	13.0	Photometry
Hematocrit	portion	0.42	0.03	Calculated
Mean cell volume	fl	89.9	4.15	Calculated
Mean cell hemoglobin	pg	30.4	58.1	Calculated
**Other**				
Calcium	mmol/L	2.36	0.08	Biochemical
Urate	mmol/L	0.27	0.07	Biochemical
Urea	mmol/L	0.72	0.03	NMR

Abbreviations: HDL = high-density lipoprotein, IDL = intermediate-density lipoprotein, LDL = low-density lipoprotein, NMR = nuclear magnetic resonance spectroscopy, VLDL = very-low-density lipoprotein

Supplementary Table 2. Logistic regression models (1-3)* predicting fatty liver (FL_{All}) with hsa-miR-122-5p or -885-5p and known risk factors and biomarkers of FL in different subgroups of Young Finns Study.

Study group	MODEL 1*		MODEL 2		MODEL 3	
	miR-122	miR-885	miR-122	miR-885	miR-122	miR-885
**All subjects (**NL n=724, FL_{All} n=147)						
n	703	871	701	868	632	781
p-value	$1.26*10^{-14}$	$3.91*10^{-6}$	$1.29*10^{-8}$	0.002	$6.84*10^{-5}$	0.131
OR	2.44	1.55	2.07	1.41	1.78	1.23
95% CI	1.96-3.09	1.29-1.86	1.62-2.68	1.13-1.77	1.35-2.38	0.94-1.60
**Women** (NL n =438, FL_{All} n = 38)						
n	358	476	356	473	321	425
p-value	$6.41*10^{-4}$	0.034	0.008	0.031	0.010	0.136
OR	2.03	1.44	1.85	1.54	2.02	1.44
95% CI	1.36-3.09	1.03-2.03	1.18-3.00	1.05-2.31	1.20-3.56	0.90-2.37
**Men** (NL n = 286, FL_{All} n = 109)						
n	345	395	345	395	311	356
p-value	$6.17*10^{-10}$	0.012	$5.01*10^{-7}$	0.029	0.001	0.471
OR	2.49	1.33	2.18	1.33	1.77	1.12
95% CI	1.88-3.36	1.07-1.68	1.62- 2.98	1.03-1.73	1.26-2.51	0.83-1.53
**Normal weight; BMI ≤ 25** (NL n = 364, FL_{All} n = 18)						
n	298	382	298	382	273	351
p-value	0.001	0.012	0.002	0.080	0.003	0.125
OR	2.59	1.88	2.45	1.64	3.1	1.76
95% CI	1.51-4.65	1.16-3.11	1.42-3.14	0.96-1.27	1.54-7.0	0.89-3.82
**Overweight or obese; BMI > 25** (NL n = 357, FL_{All} n = 134)						
n	403	486	403	486	359	430
p-value	$4.06*10^{-9}$	$1.18*10^{-4}$	$2.22*10^{-6}$	0.014	0.003	0.191
OR	2.15	1.52	1.92	1.35	1.57	1.2
95% CI	1.68-2.79	1.23-1.88	1.47-2.53	1.06-1.71	1.17-2.13	0.91-1.59
**Moderate alcohol usage; < 20 g of ethanol per day** (NL n =623, FL_{All} n = 95)						
n	571	718	569	715	542	681
p-value	$4.98*10^{-10}$	$8.82*10^{-4}$	$4.71*10^{-6}$	0.057	0.001	0.523
OR	2.35	1.46	2.03	1.30	1.75	1.10
95% CI	1.81-3.09	1.17-1.82	1.51-2.77	0.99-1.70	1.28-2.44	0.82-1.49
**Excess alcohol usage; ≥ 20 g of ethanol per day** (NL n = 70, FL_{All} n = 38)						
n	98	108	98	108	90	100
p-value	$2.60*10^{-4}$	0.004	0.002	0.006	0.059	0.016
OR	2.75	1.95	2.64	2.07	2.03	2.08
95% CI	1.66-4.95	1.27-3.15	1.51-5.12	1.26-3.61	1.02-4.59	1.18-3.94

MODEL 1: *Stepwise logistic regression model predicting liver status with hsa-miR-122-5p or hsa-miR-885-5p (one by one forced into model).

MODEL 2: Model 1+ age, sex (sex not included in the sex specific analysis) and BMI.

MODEL 3: Model 2 + alcohol consumption, waist circumference, apolipoprotein B levels, triglycerides, insulin levels, systolic blood pressure, smoking, and physical activity index. Model involves all the explanatory variables that have been previously associated with liver status in Young Finns Study [see ref. 6] excluding liver enzymes due to high correlation with studied miRNAs.

Abbreviations: NL = normal liver, FL_{All} = all subjects with fatty liver.

Supplementary Table 3. Continuous net reclassification improvement (NRI) of risk stratification of fatty liver after adding hsa-miR-122-5p and hsa-885-5p to the base model* of conventional risk factors.

Subgroup/modeling	AUC	NRI (95% CI)	p-value
**Men** (NL n=226, FL_{All}, n=85)			
Model*	0.849	Reference	Reference
+ hsa-miR-122-5p	0.850	0.188 (-0.059 - 0.436)	0.14
+ hsa-miR-885-5p	0.849	0.059 (-0.190 - 0.308)	0.64
+ hsa-miR-122-5p and hsa-miR-885-5p	0.853	0.383 (0.142 - 0.624)	0.0019
**Women** (NL n=298, FL_{All}, n=23)			
Model	0.933	Reference	Reference
+ hsa-miR-122-5p	0.937	0.291 (-0.115 - 0.696)	0.16
+ hsa-miR-885-5p	0.933	-0.097 (-0.518 - 0.324)	0.65
+ hsa-miR-122-5p and hsa-miR-885-5p	0.936	0.445 (0.068 - 0.821)	0.021
**BMI ≤ 25** (NL n=260, FL_{All}, n=13**)**			
Model	0.901	Reference	Reference
+ hsa-miR-122-5p	0.905	0.169 (-0.373 - 0.712)	0.54
+ hsa-miR-885-5p	0.899	0.162 (-0.381 - 0.704)	0.56
+ hsa-miR-122-5p and hsa-miR-885-5p	0.906	0.200 (-0.343 - 0.743)	0.47
**BMI > 25** (NL n=264, FL_{All}, n=95)			
Model	0.857	Reference	Reference
+ hsa-miR-122-5p	0.860	0.112 (-0.120 - 0.345)	0.34
+ hsa-miR-885-5p	0.858	0.067 (-0.1672 - 0.301)	0.58
+ hsa-miR-122-5p and hsa-miR-885-5p	0.860	0.131 (-0.102 - 0.364)	0.27

***Statistical model:** Stepwise regression model consisting of variables associated with FL in the Young Finns Study [see ref 6]. **Abbreviations:** AUC = area under curve, NL = normal liver, FL_{All} = all subjects with fatty liver**,** ALT = Alanine aminotransferase, GT = gamma-glutamyltransferase.

Supplementary Table 4. Associations of hsa-miR-122-5p and hsa-miR-885-5p with lipoprotein subclasses form the NMR lipo window. In the regression model* the Betas (β) indicate the standard deviation (SD) change of the metabolite levels per increase of one SD of miRNA levels. Significant p-values are indicated in bold.

	Hsa-miR-122-5p		Hsa-miR-885-5p	
**Metabolite**	**p-value**	**β (95%CI)**	**p-value**	**β (95%CI)**
**Particles**				
XXL VLDL	0.815	-0.008 (-0.077-0.060)	0.649	-0.014 (-0.073-0.045)
XL VLDL	0.224	0.039 (-0.024-0.101)	0.505	0.019 (-0.036-0.073)
L VLDL	0.451	0.026 (-0.042-0.093)	0.297	0.032 (-0.028-0.092)
M VLDL	0.343	0.035 (-0.037-0.107)	0.205	0.038 (-0.021-0.097)
S VLDL	0.083	0.062 (-0.008-0.132)	0.183	0.040 (-0.019-0.098)
XS VLDL	**0.003**	**0.112 (0.038-0.185)**	0.650	0.015 (-0.051-0.082)
IDL	**0.009**	**0.102 (0.025-0.178)**	0.798	0.009 (-0.058-0.076)
L LDL	**0.039**	**0.082 (0.004-0.159)**	0.998	0.000 (-0.067-0.067)
M LDL	0.085	0.066 (-0.009-0.141)	0.962	-0.002 (-0.068-0.065)
S LDL	0.144	0.055 (-0.019-0.129)	0.673	-0.014 (-0.080-0.052)
XL HDL	0.231	-0.043 (-0.112-0.027)	**0.038**	**-0.060 (-0.117--0.003)**
L HDL	0.142	-0.052 (-0.120-0.017)	0.102	-0.048 (-0.105-0.009)
M HDL	0.299	-0.043 (-0.125-0.038)	0.085	-0.061 (-0.130-0.008)
S HDL	0.396	-0.033 (-0.110-0.044)	0.132	-0.051 (-0.118-0.015)
**Lipids**				
XXL VLDL	0.232	0.040 (-0.026-0.106)	0.870	-0.005 (-0.062-0.053)
XL VLDL	0.329	0.032 (-0.032-0.095)	0.540	0.018 (-0.038-0.074)
L VLDL	0.398	0.030 (-0.040-0.101)	0.352	0.028 (-0.031-0.087)
M VLDL	0.302	0.038 (-0.034-0.110)	0.168	0.042 (-0.018-0.101)
S VLDL	0.083	0.063 (-0.008-0.133)	0.203	0.038 (-0.020-0.097)
XS VLDL	**0.004**	**0.110 (0.036-0.184)**	0.688	0.014 (-0.054-0.081)
IDL	**0.010**	**0.101 (0.024-0.178)**	0.777	0.010 (-0.058-0.077)
L LDL	**0.043**	**0.080 (0.003-0.158)**	0.953	0.002 (-0.065-0.070)
M LDL	0.090	0.065 (-0.010-0.140)	0.971	0.001 (-0.065-0.068)
S LDL	0.087	0.067 (-0.009-0.143)	0.750	-0.011 (-0.077-0.055)
XL HDL	0.239	-0.043 (-0.114-0.028)	**0.032**	**-0.064 (-0.122--0.006)**
L HDL	0.111	-0.056 (-0.125-0.013)	0.084	-0.050 (-0.107-0.007)
M HDL	0.248	-0.056 (-0.125-0.013)	0.103	-0.058 (-0.127-0.012)
S HDL	0.545	-0.024 (-0.100-0.053)	0.212	-0.043 (-0.110-0.024)
**Phospholipids**				
XXL VLDL	0.357	0.031 (-0.035-0.097)	0.948	0.002 (-0.056-0.060)
XL VLDL	0.129	0.050 (-0.014-0.114)	0.702	0.011 (-0.046-0.068)
L VLDL	0.304	0.037 (-0.034-0.108)	0.321	0.030 (-0.029-0.089)
M VLDL	0.227	0.037 (-0.034-0.108)	0.161	0.042 (-0.017-0.101)
S VLDL	0.054	0.070 (-0.001-0.142)	0.197	0.039 (-0.020-0.098)
XS VLDL	**0.003**	**0.114 (0.038-0.190)**	0.361	0.031 (-0.036-0.098)
IDL	**0.026**	**0.090 (0.011-0.169)**	0.651	0.016 (-0.052-0.084)
L LDL	**0.040**	**0.082 (0.004-0.159)**	0.905	0.004 (-0.063-0.072)
M LDL	0.079	0.066 (-0.008-0.140)	0.966	-0.001 (-0.067-0.064)
XL HDL	0.201	-0.044 (-0.113-0.024)	**0.049**	**-0.056 (-0.112-0.000)**
L HDL	0.196	-0.046 (-0.114-0.023)	0.093	-0.050 (-0.108-0.008)
M HDL	0.271	-0.046 (-0.127-0.035)	0.094	-0.059 (-0.128-0.010)
**Triglycerides**				
Total	0.116	0.058 (-0.014-0.129)	0.396	0.026 (-0.034-0.085)
VLDL	0.197	0.047 (-0.024-0.117)	0.295	0.031 (-0.027-0.090)
XXL VLDL	0.173	0.046 (-0.020-0.112)	0.783	-0.008 (-0.066-0.050)
XL VLDL	0.339	0.031 (-0.033-0.095)	0.550	0.017 (-0.039-0.073)
L VLDL	0.521	0.023 (-0.048-0.094)	0.347	0.028 (-0.031-0.088)
M VLDL	0.473	0.026 (-0.045-0.098)	0.277	0.033 (-0.026-0.092)
S VLDL	0.102	0.058 (-0.012-0.129)	0.205	0.037 (-0.021-0.096)
XS VLDL	**0.010**	**0.096 (0.023-0.169)**	0.414	0.026 (-0.036-0.088)
IDL	**0.019**	**0.091 (0.015-0.166)**	0.787	0.009 (-0.056-0.074)
XL HDL	0.090	0.071 (-0.011-0.154)	0.660	-0.015 (-0.084-0.053)
S HDL	0.058	0.071 (-0.002-0.143)	0.213	0.038 (-0.022-0.099)
**Cholesterol**				

Total	0.185	0.053 (-0.025-0.131)	0.671	-0.015 (-0.083-0.054)
LDL	0.063	0.072 (-0.004-0.151)	0.957	0.002 (-0.065-0.069)
HDL	0.099	-0.062 (-0.136-0.012)	**0.030**	**-0.069 (-0.131--0.007)**
L VLDL	0.225	0.043 (-0.026-0.113)	0.248	0.034 (-0.024-0.092)
M VLDL	0.143	0.052 (-0.017-0.120)	0.106	0.050 (-0.011-0.110)
S VLDL	0.051	0.069 (0.000-0.138)	0.197	0.040 (-0.021-0.102)
IDL	**0.015**	**0.097 (0.019-0.175)**	0.667	0.015 (-0.053-0.083)
L LDL	0.050	0.078 (0.000-0.156)	0.853	0.006 (-0.061-0.074)
M LDL	0.069	0.071 (-0.005-0.148)	0.898	0.006 (-0.061-0.074)
S LDL	0.090	0.066 (-0.010-0.143)	0.856	-0.006 (-0.073-0.060)
XL HDL	0.326	-0.037 (-0.112-0.037)	**0.031**	**-0.067 (-0.128--0.006)**
L HDL	0.078	-0.062 (-0.131-0.007)	0.053	-0.056 (-0.113-0.001)
M HDL	0.174	-0.057 (-0.138-0.025)	0.061	-0.066 (-0.135-0.003)
**Cholesterol esters**				
L VLDL	0.261	0.040 (-0.030-0.111)	0.148	0.043 (-0.015-0.101)
M VLDL	0.101	0.059 (-0.012-0.130)	0.099	0.052 (-0.010-0.114)
L LDL	0.051	0.077 (0.000-0.155)	0.816	0.008 (-0.059-0.075)
M LDL	0.067	0.071 (-0.005-0.148)	0.816	0.008 (-0.058-0.074)
XL HDL	0.337	-0.037 (-0.112-0.038)	**0.026**	**-0.070 (-0.131--0.008)**
L HDL	0.060	-0.067 (-0.138-0.003)	0.062	-0.055 (-0.112-0.003)
M HDL	0.138	-0.062 (-0.143-0.020)	**0.048**	**-0.070 (-0.139--0.001)**
**Free cholesterol**				
L VLDL	0.301	0.034 (-0.031-0.099)	0.453	0.022 (-0.036-0.080)
M VLDL	0.184	0.049 (-0.023-0.121)	0.137	0.045 (-0.014-0.105)
S VLDL	**0.017**	**0.086 (0.016-0.157)**	0.185	0.040 (-0.019-0.100)
IDL	**0.013**	**0.099 (0.021-0.177)**	0.409	0.029 (-0.039-0.097)
L LDL	**0.034**	**0.085 (0.007-0.164)**	0.811	0.008 (-0.061-0.078)
XL HDL	0.290	-0.039 (-0.111-0.033)	**0.026**	**-0.067 (-0.126--0.008)**
L HDL	0.237	-0.041 (-0.108-0.027)	0.090	-0.048 (-0.104-0.007)
M HDL	0.464	-0.031 (-0.113-0.051)	0.199	-0.046 (-0.115-0.024)
**Apolipoproteins**				
ApoA1	0.427	-0.033 (-0.113-0.048)	0.062	-0.065 (-0.133-0.003)
ApoB	**0.020**	**0.086 (0.014-0.158)**	0.428	0.026 (-0.038-0.090)
ApoA1/ApoB	**0.006**	**0.095 (0.027-0.163)**	0.110	0.048 (-0.011-0.107)

***Statistical model:** Stepwise linear (AIC) regression model with individual miRNA (one by one forced into model) age, sex, BMI, liver status, ALT, AST and GT are used to predict NMR metabolite levels. **Note:** individuals with ALT, AST and GT levels over the Finnish reference ranges were removed from the analysis. **Abbreviations:** XL=extra large, L=large, M=medium, S=small, XS=extra small, HDL=high density lipoprotein, LDL=low density lipoprotein, IDL=intermediate density lipoprotein, VLDL=very low density lipoprotein. ALT=alanine aminotransferase AST= aspartate aminotransferase, GT=gamma-glutamyltransferase.

Supplementary Table 5. Associations between levels of hsa-miR-885-5p and its *in silico* predicted mRNA target expression levels from transcriptomics analysis. In the regression model the Betas (b) presents the standard error (SD) increment of the target mRNA levels per increase of one SD of miRNA levels.

		Spearman correlation			Linear regression model*					
**Gene ID**	**Accesion ID**	**n**	**p-value**	**r**	**n**	**p-value**	**β**	**CI(95%)**		
GABARAP	NM_007278.1	740	0.032	-0.079	712	0.002	-0.120	-0.194	–	-0.046
ARSA	NM_000487.3	740	0.022	-0.084	712	0.005	-0.111	-0.188	–	-0.035
AHNAK	NM_024060.2	740	3.20E-05	0.152	712	0.013	0.094	0.020	–	0.168
RSPH3	NM_031924.3	740	0.002	-0.111	712	0.014	-0.094	-0.169	–	-0.019
OSBPL2	NM_144498.1	740	1.00E-04	-0.143	712	0.015	-0.095	-0.171	–	-0.019
UQCC	NM_199487.1	740	0.001	0.126	712	0.015	0.095	0.019	–	0.172
HNRNPL	NM_001005335.1	740	0.032	0.079	712	0.018	0.091	0.016	–	0.166
TRO	NM_001039705.1	740	4.60E-05	0.149	712	0.019	0.092	0.015	–	0.169
FCRL6	NM_001004310.1	740	0.041	0.075	712	0.022	0.093	0.014	–	0.172
APOL3	NM_030644.1	740	0.001	0.118	712	0.022	0.088	0.013	–	0.164
PPP2R1A	NM_014225.3	740	0.005	0.102	712	0.028	0.087	0.010	–	0.165
AGPAT4	NM_001012734.1	740	0.004	0.104	712	0.066	0.071	-0.004	–	0.146
RNPEPL1	NM_018226.3	740	0.012	0.092	712	0.084	0.066	-0.009	–	0.142
DHRS4	NM_021004.2	740	0.009	0.096	712	0.110	0.064	-0.014	–	0.143
HBP1	NM_012257.3	740	0.014	-0.090	712	0.152	-0.055	-0.131	–	0.020
YWHAE	NM_006761.3	740	0.007	0.098	712	0.153	0.057	-0.021	–	0.134
PVRL2	NM_002856.1	740	0.002	-0.115	712	0.182	-0.053	-0.131	–	0.025
VPS37C	NM_017966.4	740	0.032	-0.079	712	0.186	-0.050	-0.124	–	0.024
HINT3	NM_138571.4	740	0.036	-0.077	712	0.188	-0.053	-0.133	–	0.026
ZNF562	NM_017656.1	740	0.019	0.086	712	0.189	0.052	-0.025	–	0.129
AHNAK	NM_001620.1	740	0.005	0.102	712	0.237	0.045	-0.030	–	0.119
RNASEL	NM_021133.2	740	0.024	-0.083	712	0.242	-0.044	-0.118	–	0.030
FPR2	NM_001462.3	740	0.003	-0.108	712	0.271	-0.042	-0.116	–	0.033
SPAG9	NM_003971.3	740	0.015	-0.090	712	0.287	-0.193	-0.115	–	0.034
KIAA1598	NM_018330.3	740	0.003	0.108	712	0.288	0.042	-0.035	–	0.118
SPAG9	NM_172345.1	740	0.034	-0.078	712	0.364	-0.035	-0.112	–	0.041
RUSC1	NM_014328.2	740	0.043	0.074	712	0.374	0.035	-0.042	–	0.111
TMEM71	NM_144649.1	740	0.001	-0.125	712	0.376	-0.034	-0.108	–	0.041
TNFSF8	NM_001244.2	740	0.023	-0.084	712	0.388	-0.034	-0.112	–	0.044
TBCD	NM_005993.3	740	0.020	0.086	712	0.413	0.032	-0.045	–	0.109
TPMT	NM_000367.2	740	0.049	0.072	712	0.422	0.031	-0.045	–	0.108
ZXDC	NM_025112.3	740	0.016	-0.088	712	0.477	-0.028	-0.105	–	0.049
SLC2A6	NM_017585.2	740	0.004	0.105	712	0.609	0.020	-0.056	–	0.096
MXD1	NM_002357.2	740	0.003	-0.107	712	0.640	-0.017	-0.090	–	0.055
FPR2	NM_001462.3	740	0.009	-0.096	712	0.640	-0.018	-0.093	–	0.057
ADD1	NM_001119.3	740	0.040	0.075	712	0.650	-0.018	-0.093	–	0.058
EIF1AX	NM_001412.3	740	0.050	-0.072	712	0.650	-0.018	-0.095	–	0.059
PGS1	NM_024419.3	740	0.018	-0.087	712	0.667	-0.016	-0.092	–	0.059
AQP9	NM_020980.2	740	0.016	-0.089	712	0.697	0.014	-0.058	–	0.087
KLHL6	NM_130446.1	740	0.015	-0.089	712	0.703	-0.015	-0.092	–	0.062
ZC3HAV1	NM_020119.3	740	0.050	-0.072	712	0.774	-0.011	-0.085	–	0.063
RPGR	NM_001023582.1	740	0.020	-0.086	712	0.783	-0.011	-0.086	–	0.065
RBM33	NM_001008408.1	740	0.004	-0.106	712	0.898	-0.005	-0.081	–	0.071
USP9X	NM_004652.3	740	0.002	-0.115	712	0.926	0.004	-0.071	–	0.078
PROK2	NM_021935.2	740	3.21E-04	-0.132	712	0.930	0.003	-0.066	–	0.073
SPAG9	NM_003971.3	740	0.015	-0.090	712	0.949	-0.002	-0.078	–	0.073
TMEM19	NM_018279.2	740	0.024	0.083	712	0.993	0.000	-0.076	–	0.076
RNASEL	NM_021133.2	740	0.017	-0.087	712	0.994	0.000	-0.073	–	0.074

*__Model:__ Stepwise linear regression model with hsa-miR-885-5p (forced in the model), age, sex, BMI, liver status, alanine aminotransferase (ALT), aspartate aminotransferase (AST) and gamma-glutamyltransferase (GT). **Note:** Individuals with ALT, AST or GT levels over the Finnish reference ranges were discarded from the analysis.

Supplementary Table 6. Associations between levels of hsa-miR-122-5p and its *in silico* predicted mRNA target expression levels from transcriptomics analysis. In the regression model the betas (b) presents the standard deviation (SD) increment of the target mRNA levels per increase of one SD of miRNA levels.

		Spearman correlation			Linear regression model*			
**Gene ID**	**Accession ID**	**n**	**p-value**	**r**	**n**	**p-value**	**β**	**CI(95%)**
SUSD1	NM_022486.3	616	0.001	0.138	586	0.042	0.085	0.003 – 0.167
PSEN2	NM_012486.1	616	0.001	0.128	586	0.143	0.062	-0.021 – 0.144
CS	NM_004077.2	616	0.002	0.122	586	0.005	0.131	0.041 – 0.222
TRAK1	NM_014965.2	616	0.002	0.123	586	0.009	0.122	0.031 – 0.213
CANX	NM_001024649.1	616	0.003	0.118	586	0.037	0.097	0.006 – 0.188
RBM28	NM_018077.1	616	0.003	0.120	586	0.054	0.083	-0.001 – 0.166
PTP4A1	NM_003463.3	616	0.004	0.114	586	0.011	0.113	0.026 – 0.200
NONO	NM_007363.3	616	0.004	0.117	586	0.736	0.016	-0.075 – 0.106
GBP4	NM_052941.2	616	0.006	0.111	586	0.139	0.063	-0.020 – 0.146
CS	NM_004077.2	616	0.006	0.111	586	0.141	0.066	-0.022 – 0.155
UBQLN1	NM_053067.1	616	0.007	0.108	586	0.135	0.065	-0.020 – 0.150
FBXO7	NM_012179.3	616	0.007	0.109	586	0.947	-0.003	-0.092 – 0.086
CANX	NM_001024649.1	616	0.008	0.107	586	0.202	0.055	-0.029 – 0.138
RALGAPA1	NM_194301.2	616	0.008	-0.107	586	0.311	-0.043	-0.127 – 0.041
TTYH3	NM_025250.2	616	0.008	0.107	586	0.373	0.040	-0.048 – 0.128
PKM2	NM_182471.1	616	0.008	0.107	586	0.614	-0.023	-0.113 – 0.067
TCN2	NM_000355.2	616	0.009	0.105	586	0.494	0.030	-0.056 – 0.115
DOPEY2	NM_005128.2	616	0.010	0.103	586	0.171	0.063	-0.027 – 0.154
FAM84B	NM_174911.3	616	0.010	0.104	586	0.626	-0.022	-0.113 – 0.068
MED26	NM_004831.3	616	0.011	0.102	586	0.060	0.080	-0.003 – 0.163
ZDHHC24	NM_207340.1	616	0.011	0.102	586	0.341	0.044	-0.046 – 0.134
SELENBP1	NM_003944.2	616	0.011	0.103	586	0.527	-0.028	-0.114 – 0.059
OSBP2	NM_030758.3	616	0.011	0.103	586	0.875	0.007	-0.083 – 0.097
RBM47	NM_019027.3	616	0.012	-0.101	586	0.836	0.009	-0.075 – 0.092
EIF1AX	NM_001412.3	616	0.014	-0.099	586	0.016	-0.111	-0.201 – -0.021
HMGN1	NM_004965.6	616	0.015	-0.098	586	0.169	-0.059	-0.144 – 0.025
PIP4K2A	NM_005028.4	616	0.015	0.098	586	0.328	0.046	-0.046 – 0.138
ABCC5	NM_001023587.1	616	0.015	-0.098	586	0.351	-0.040	-0.124 – 0.044
LAMC1	NM_002293.2	616	0.016	0.097	586	0.018	0.103	0.018 – 0.188
LUC7L	NM_018032.3	616	0.016	-0.097	586	0.130	-0.064	-0.147 – 0.019
TGM3	NM_003245.2	616	0.016	-0.097	586	0.182	-0.054	-0.133 – 0.025
PRPF38B	NM_018061.1	616	0.017	-0.096	586	0.922	-0.004	-0.090 – 0.082
FAM172A	NM_032042.4	616	0.018	-0.095	586	0.133	-0.068	-0.157 – 0.021
FBXO7	NM_001033024.1	616	0.018	0.095	586	0.534	0.027	-0.058 – 0.112
SLC1A5	NM_005628.1	616	0.019	0.094	586	0.482	-0.032	-0.120 – 0.057
SORT1	NM_002959.4	616	0.020	0.094	586	0.031	0.099	0.009 – 0.189
NEXN	NM_144573.1	616	0.020	0.094	586	0.411	0.035	-0.048 – 0.118
GUCY1A3	NM_000856.2	616	0.022	0.093	586	0.634	0.021	-0.065 – 0.106
SLC25A5	NM_001152.1	616	0.023	0.091	586	0.114	0.067	-0.016 – 0.150
FNDC3A	NM_001079673.1	616	0.023	-0.092	586	0.276	-0.047	-0.132 – 0.038
TMEM39B	NM_018056.1	616	0.024	0.091	586	0.529	0.029	-0.060 – 0.118
TIAL1	NM_003252.3	616	0.025	-0.090	586	0.782	-0.013	-0.103 – 0.078
RUNX2	NM_001024630.1	616	0.025	-0.091	586	0.929	0.004	-0.083 – 0.091
OSBPL10	NM_017784.3	616	0.028	-0.089	586	0.267	-0.050	-0.138 – 0.038
BCL2L2	NM_004050.2	616	0.029	0.088	586	0.014	0.114	0.023 – 0.205
MAF1	NM_032272.3	616	0.029	0.088	586	0.603	-0.022	-0.106 – 0.062
PARP3	NM_005485.3	616	0.030	0.088	586	0.382	0.037	-0.046 – 0.120
CTDSPL	NM_005808.2	616	0.030	0.087	586	0.568	0.024	-0.058 – 0.106
KLHL20	NM_014458.3	616	0.030	-0.088	586	0.782	-0.012	-0.101 – 0.076
CHD7	NM_017780.2	616	0.031	-0.087	586	0.747	0.015	-0.075 – 0.104
CCDC69	NM_015621.2	616	0.032	0.087	586	0.013	0.106	0.023 – 0.190
TRAK1	NM_001042646.1	616	0.032	0.086	586	0.013	0.109	0.023 – 0.194
CCDC43	NM_144609.1	616	0.032	0.086	586	0.014	0.102	0.021 – 0.182
CS	NM_004077.2	616	0.032	0.086	586	0.068	0.078	-0.006 – 0.161
MEA1	NM_014623.2	616	0.032	0.086	586	0.146	0.062	-0.021 – 0.145

LUZP1	NM_033631.2	616	0.032	0.086	586	0.456	0.032	-0.053	−	0.117
LCMT1	NM_016309.2	616	0.033	0.086	586	0.014	0.105	0.021	−	0.188
ZC3H10	NM_032786.1	616	0.033	-0.086	586	0.039	-0.090	-0.175	−	-0.005
RGL1	NM_015149.2	616	0.033	-0.086	586	0.238	-0.055	-0.147	−	0.036
EIF1AX	NM_001412.3	616	0.034	-0.085	586	0.128	-0.066	-0.151	−	0.019
SEC22C	NM_032970.2	616	0.034	0.085	586	0.656	0.021	-0.070	−	0.111
KLC1	NM_005552.3	616	0.035	0.085	586	0.105	0.069	-0.014	−	0.153
FAHD1	NM_001018104.1	616	0.035	0.085	586	0.806	0.011	-0.074	−	0.096
OCRL	NM_001587.3	616	0.036	0.085	586	0.692	0.019	-0.074	−	0.112
ARSB	NM_000046.2	616	0.038	0.084	586	0.005	0.118	0.036	−	0.201
PROK2	NM_021935.2	616	0.038	-0.083	586	0.503	0.027	-0.053	−	0.108
CD40LG	NM_000074.2	616	0.039	-0.083	586	0.651	-0.019	-0.102	−	0.064
GALNT10	NM_198321.2	616	0.042	0.082	586	0.042	0.097	0.004	−	0.191
JMJD1C	NM_004241.2	616	0.042	-0.082	586	0.817	0.010	-0.075	−	0.095
FAM104A	NM_032837.1	616	0.043	0.081	586	0.308	-0.046	-0.135	−	0.042
PPAPDC1B	NM_032483.2	616	0.044	0.081	586	0.041	0.096	0.004	−	0.187
ATP5A1	NM_004046.4	616	0.044	0.081	586	0.049	0.092	0.001	−	0.184
LGR6	NM_001017403.1	616	0.044	0.081	586	0.075	0.077	-0.008	−	0.162
NRSN2	NM_024958.1	616	0.045	0.081	586	0.927	0.004	-0.089	−	0.098
SHCBP1	NM_024745.2	616	0.047	0.080	586	0.020	0.105	0.017	−	0.194
SLC7A5	NM_003486.5	616	0.047	0.080	586	0.651	-0.021	-0.112	−	0.070
MLLT10	NM_004641.2	616	0.050	-0.079	586	0.634	0.021	-0.067	−	0.110

[*] **Model:** Stepwise linear regression model with hsa-miR-122-5p (forced in the model), age, sex, BMI, liver status, alanine aminotransferase (ALT), aspartate aminotransferase (AST) and gamma-glutamyltransferase (GT). **Note:** Individuals with ALT, AST or GT levels over the Finnish reference ranges were discarded from the analysis.
